# Dual level dengue diagnosis using lightweight multilayer perceptron with XAI in fog computing environment and rule based inference

**DOI:** 10.1038/s41598-025-98365-6

**Published:** 2025-05-13

**Authors:** Deepika R., Pradeep Kumar T.S.

**Affiliations:** School of Computer Science and Engineering, Vellore Institute of Technology, Chennai, 600 127 India

**Keywords:** Diseases, Health care, Medical research

## Abstract

Over the last fifty years, arboviral infections have made an unparalleled contribution to worldwide disability and morbidity. Globalization, population growth, and unplanned urbanization are the main causes. Dengue is regarded as the most significant arboviral illness among them due to its prior dominance in growth. The dengue virus is mostly transmitted to humans by Aedes mosquitoes. The human body infected with dengue virus (DenV) will experience certain adverse impacts. To keep the disease under control, some of the preventative measures implemented by different countries need to be updated. Manual diagnosis is typically employed, and the accuracy of the diagnosis is assessed based on the experience of the healthcare professionals. Because there are so many patients during an outbreak, incompetence also happens. Remote monitoring and massive data storage are required. Though cloud computing is one of the solutions, it has a significant latency, despite its potential for remote monitoring and storage. Also, the diagnosis should be made as quickly as possible. The aforementioned issue has been resolved with fog computing, which significantly lowers latency and facilitates remote diagnosis. This study especially focuses on incorporating machine learning and deep learning techniques in the fog computing environment to leverage the overall diagnostic efficiency of dengue by promoting remote diagnosis and speedy treatment. A dual-level dengue diagnosis framework has been proposed in this study. Level-1 diagnosis is based on the symptoms of the patients, which are sent from the edge layer to the fog. Level-1 diagnosis is done in the fog to manage the storage and computation issues. An optimized and normalized lightweight MLP has been proposed along with preprocessing and feature reduction techniques in this study for the Level-1 Diagnosis in the fog computing environment. Pearson Correlation coefficient has been calculated between independent and target features to aid in feature reduction. Techniques like K-fold cross-validation, batch normalization, and grid search optimization have been used for increasing the efficiency. A variety of metrics have been computed to assess the effectiveness of the model. Since the suggested model is a “black box,” explainable artificial intelligence (XAI) tools such as SHAP and LIME have been used to help explain its predictions. An exceptional accuracy of 92% is attained with the small dataset using the proposed model. The fog layer sends the list of probable cases to the edge layer. Also, a precision of 100% and an F1 score of 90% have been attained using the proposed model. The list of probable cases is sent from the fog layer to the edge layer, where Level-2 Diagnosis is carried out. Level-2 diagnosis is based on the serological test report of the suspected patients of the Level-1 diagnosis. Level-2 diagnosis is done at the edge using the rule-based inference method. This study incorporates dual-level diagnosis, which is not seen in recent studies. The majority of investigations end at Level 1. However, this study minimizes incorrect treatment and fatality rates by using dual-level diagnosis and assisting in confirmation of the disease.

## Introduction

One of the most prevalent mosquito-borne diseases, dengue virus infection affects both tropical and subtropical areas and can infect up to 100–400 million people year worldwide (WHO, 2021). The worldwide dengue epidemic’s distribution shows that dengue infection outbreaks are occurring everywhere in the world. A new WHO (World Health Organization) assessment states that DENV (Dengue Virus) is now a significant virus-caused disease, second only to COVID-19, due to the rising number of infected individuals in 2020 (WHO, 2021). The Philippines, Vietnam, India, Colombia, and Brazil were found to have the greatest rates of DENV infection among these nations. The rapid urbanization and poor infrastructure design that may result in ineffective vector control management are two of the primary causes of the spread of diseases carried by mosquitoes worldwide^1^. Mosquitoes carrying the Dengue virus are the source of dengue. The mosquitoes that cause dengue are Aedes aegypti and Aedes albopictus. Both laboratory and symptomatic diagnostics are used to diagnose dengue. Symptoms of a dengue virus infection typically include a high fever, rash, erythema, headache, vomiting, and other symptoms^2^. The symptoms are used to diagnose probable instances of dengue. To confirm dengue cases, laboratory tests such as the NS1(Non-structural protein 1) test, IgG (Immunoglobulin G) test, and IgM (Immunoglobulin M) test are performed. These are serological tests. NS1 is the antigen test whereas IgG and IgM are antibodies test. Within 1–7 days of infection, doctors can do NS1 tests. Typically, IgM testing are performed seven days following exposure^3^.

Numerous elements, such as host genetics, immunological status, virulence, and potential pre-existing conditions, affect the pathophysiology of DENV (Dengue Virus). A range of illnesses, from mild asymptomatic dengue fever (DF) to severe dengue haemorrhagic fever (DHF) and potentially fatal dengue shock syndrome (DSS) are associated with DENV infection. The skin serves as DENV’s entry point, where the virus enters through mosquito saliva^4^. Due to the infection, antigen and antibodies are found in the blood stream of the infected person. The NS1 antigen is identified by immuno-chromatographic or immunofluorescence studies on serum and is essential to DENV replication. The immune system produces antibodies, which are proteins, to fight up an antigen attack^3^. Detection of antibodies such as IgG and IgM in serum and cerebrospinal fluid is detected by ELISA (Enzyme linked Immunosorbent assay). RT-PCR(Reverse Transcription Polymerase Chain Reaction) tests, Isothermal amplification, Antigen detection, Antibody detection, and Viral Isolation are some of the diagnostic methods prevalent in the current situation^4^.

The low level of reporting, the difficulty of disease monitoring, and misclassification mistakes are the primary disadvantages of dengue disease prediction^5^. Early detection of the virus will lessen the severity of the illness because there is currently no effective treatment. In many regions, people find it challenging to anticipate symptoms and seek medical advice due to a lack of knowledge about the virus. In some places, there is also a shortage of facilities and doctors^6^. Another reason for the requirement for an effective clinical dengue disease detection process is the limited availability of reverse transcription-polymerase chain reaction (RT-PCR) kits^5^. The development of several deep learning and machine learning methods for dengue prediction has aided doctors in reaching prompt and insightful conclusions^5^. Remote diagnosis and remote healthcare are promoted by cloud computing technologies. Though cloud computing is a solution for overcoming storage issues prevalent in storing health records and remote diagnosis, latency is a big problem with the cloud. Hence the fog layer has been introduced between the edge and the cloud layer to overcome the issue. The fog environment will make it easier to identify those who have been affected with the virus and provide them with alarm information. The alert information includes all the information required to determine the severity and the preventative measures that should be implemented^6^. The integration of fog computing guarantees smooth healthcare service delivery at any time and from any location by enabling real-time location-aware and delay-sensitive services. Numerous advantages result from the incorporation of fog computing into healthcare systems, including real-time notifications, efficient resource use, timely access to medical information, and assurance of quality of service^7^. The most crucial actions are early patient treatment and early diagnosis to control the infection^8^. These noteworthy advantages have prompted the suggested system to use fog computing as the intermediary layer. Emergency alerts and speedy diagnosis are done using the fog layer. The integrated architectures provide trustworthy patient status reporting, and the application of artificial intelligence techniques can result in accurate disease diagnosis. Also most of the works concentrated on first level of the diagnosis which helps in finding the probable cases but confirmation of the disease is done based on the serological test results. Also there is a need for a lightweight model in the fog computing environment. The main objective of this study is to develop a framework for dual-level dengue diagnosis with explainable AI, which promotes remote diagnosis and reduces the incidence of wrong treatments by finding out confirmed cases. These problems have been addressed in our proposed framework. Some of the main contributions include,

- Optimised and normalised lightweight MLP model for diagnosing probable dengue cases in the fog computing environment.
- Application of explainable AI to enhance the explainability of the proposed MLP model.
- Rule-based inference for diagnosis of the confirmed cases based on serological test results.
- Framework for dual-level diagnosis of dengue that mimics the real-world scenario.

The paper is organised in the following way. Existing works related to the topic have been discussed in the related works section, which is followed by the method section, where the steps carried out in the level-1 and level-2 diagnosis of dengue have been discussed in a detailed manner. It is then followed by the results section, which includes results obtained from various other models and the proposed model along with the performance matrices. It also includes details about the application of the explainable AI on the proposed MLP model. Various aspects of the analysis are discussed in the discussion section, which is then followed by the conclusion and future work section.

## Related works

A fog computing framework has been presented by Pravin et al.^6^ to classify the people infected with dengue based on their symptoms, send out alerts to them instantly via mobile devices, and assist physicians in determining the impact of the disease by analyzing the results and taking appropriate action in a short amount of time. Using IoT devices, the framework gathers data from local residents and processes the data to find the severity. Caio Davi et al.^9^ proposed a model that classifies patients into severe dengue and dengue fever using an artificial neural network (ANN) after using a support vector machine approach to choose the best locus classification subset. When trained on 13 important immunological Single Nucleotide Polymorphisms (SNPs) chosen using dominant or recessive models, the ANN generated mean accuracy values higher than 86%. A fog-cloud-assisted IoT-based hierarchical healthcare computing system is suggested by Sandeep Kumar Sood et al.^7^ for managing DENV infection. This system tracks and forecasts DENV infection in humans and offers a real-time remote diagnostic. Through the use of k-means clustering and fog computing, the system determines each person’s DENV infection status and instantly sends out diagnostic alerts to them at the fog layer. Additionally, employing Bayesian belief networks and artificial neural networks respectively at the cloud layer, the system monitors and forecasts the probabilistic health sensitivity of DENV-infected individuals. Jun Kit Chaw et al.^10^ had presented a study that shows how well machine learning algorithms forecast the likelihood that dengue patients would experience a shock. Neural networks, support vector machines, decision trees, and logistic regression are assessed. Finally, to maximize performance, the weak learner is additionally subjected to ensemble learning techniques such as bagging and boosting. With a 14.5% improvement over the individual decision tree, the experimental findings demonstrate that the bagging algorithm performs better than other competing approaches. Pulad Tavakolipoor et al.^11^ analyzed medical records of patients diagnosed with dengue fever (DF) and the majority of patients had fever, headache, rash, myalgia, and arthralgia when they first arrived, although some also had gastrointestinal symptoms, such as diarrhea. There were nine patients with mild haemorrhagic symptoms.

**Fig. 1:**
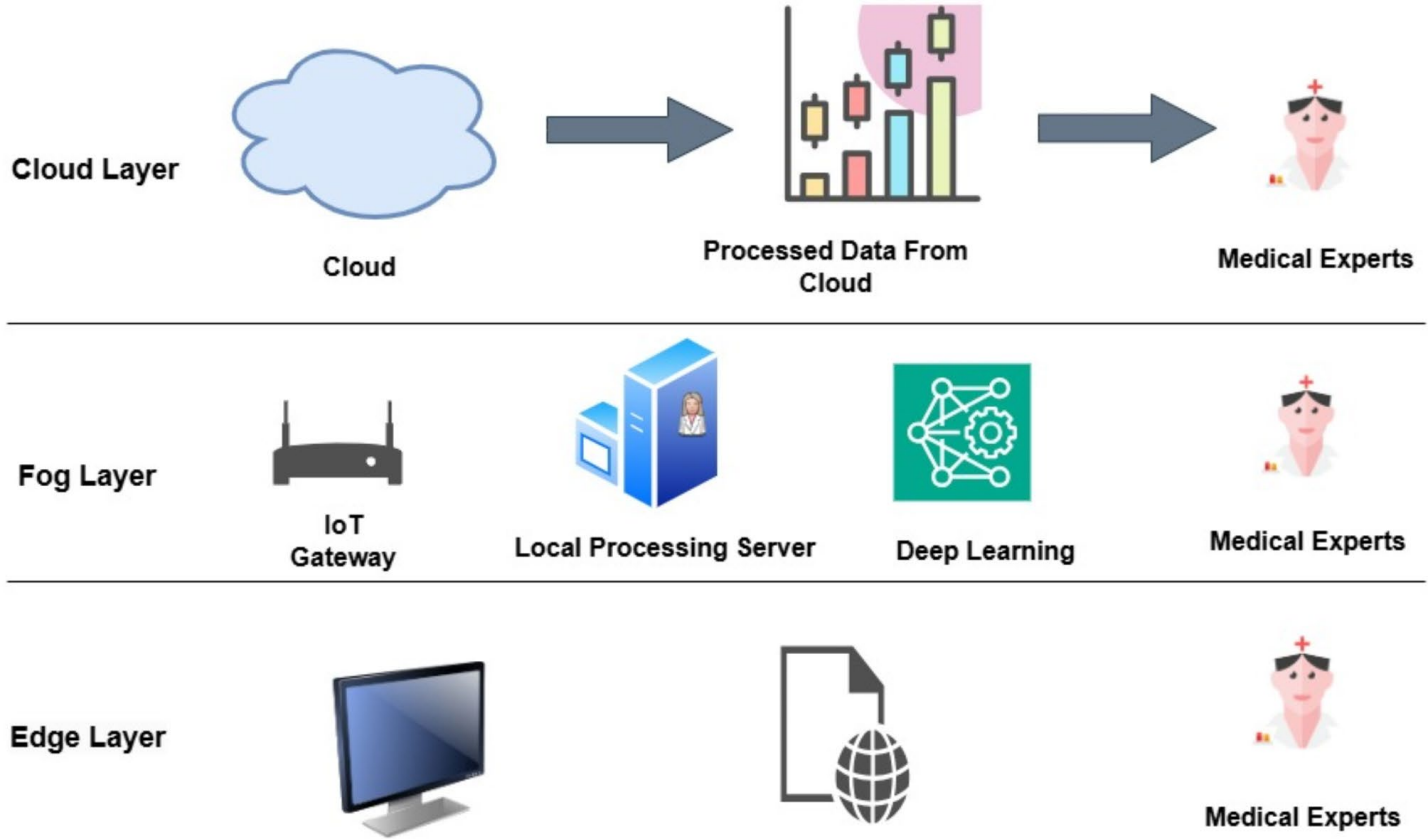
Different Layers in Smart Healthcare.

Thirteen patients experienced neurological problems. The most pertinent blood results were low platelet counts, leukopenia, and increased liver enzymes. They found that appropriate patient management requires a thorough understanding of the laboratory results and clinical complaints. William Hoyos et al.^12^ proposed a dengue decision support system based on fuzzy cognitive maps. This fuzzy cognitive map of the system is based on the signs, symptoms, and laboratory tests used in the standard diagnosis of dengue. Datasets of people with a diagnosis of dengue were used to assess the model and compare it with other approaches. With an accuracy of 89.4%, the proposed model demonstrated acceptable classification performance and was able to assess the behaviour of laboratory and clinical variables associated with dengue severity (it is an explainable method). Megha Chovatiya et al.^13^ and Ashima Kukkar et al.^14^ had proposed models for dengue diagnosis using machine learning algorithms like Recurrent Neural Network (RNN) and fog computing-enabled Weighted Random Forest respectively. Rekha Gangula et al.^15^ incorporated ensemble-based learning to predict instances of dengue more accurately and effectively. Logistic Regression Model, Decision Tree, Support Vector Machine, Naive Bayes Model, KNN (K- Nearest Neighbors) classifiers are the models used in the ensemble. Also dual level approaches have been used in many studies to increase the credibility of the diagnosis. Yang Koo Lee et al.^16^ had proposed a dual phase approach to improve the prediction of heart disease. In the first phase, the cardiac disease is predicted based on the risk factors and symptoms. In the second phase, long-term ECG recordings are used to identify HRV patterns. Claudia Yang Santos et al.^17^ had proposed a Random Forest model with 85% of accuracy to estimate the number of misdiagnosed dengue hospitalizations in Brazil. A linked dataset at the hospitalization level was created using the data. According to this estimate, dengue may have been misdiagnosed as another illness in 3.4% (13,608) of all hospitalizations in the public healthcare system between 2014 and 2020. Gaurav Gupta et al.^18^ proposed a disease prediction and diagnosis model for Dengue using sentiment analysis and machine learning algorithms. They evaluated machine learning models like Random Forest, Decision Tree, Gaussian Naive Bayes, Support Vector Machine (SVM), and K-Nearest Neighbors (KNN) and found that Random Forest performed well with a mean accuracy of 87%. Ilyas Ozer et al.^19^ proposed improved machine learning model using transfer learning which aids in the management of patients infected with arbovirus. Deepika D et al.^20,21^ proposed a unique Multilayer Perceptron for Enhanced Brownian Motion based on Dragonfly Algorithm (MLP-EBMDA) for the classification of heart disease and an enhanced unsupervised technique for feature selection. Vijayakumar V et al.^22^ suggested an intelligent system to identify and manage mosquito-borne illnesses. Wearable and Internet of Things sensors are utilized to collect the necessary data for this purpose, while fog computing has been used to evaluate, classify, and distribute medical data between patients and healthcare providers. The fuzzy k-nearest neighbour method has been used to identify the user as either sick or uninfected, and the similarity coefficient was utilized to differentiate between the various mosquito-borne diseases based on the patient’s symptoms. Furthermore, the cloud layer uses Social Network Analysis (SNA) to illustrate how diseases spread through mosquitoes. R. Arthi et al.^23^ proposed an Explainable AI (XAI) and Tiny Machine Learning (TinyML) binary classification model that uses fog computing to maximize performance for a reliable and energy-efficient healthcare decision support system. Supreet Kaur et al.^24^ proposed a machine learning-based tertiary classification technique. A tool has been utilized for data collection and analysis in order to forecast the presence of dengue infections and estimate risk levels in order to determine which group of dengue illnesses the patient has. The technology makes real-time diagnoses based on clinical and symptomatic exams. The suggested model achieves a remarkably high accuracy of over 90% along with high sensitivity and specificity values by predicting a patient’s infection levels based on the World Health Organization’s classification, which includes dengue fever, dengue haemorrhagic fever, and dengue shock syndrome. Some of the existing works focus only on the symptomatic diagnosis of dengue, which will detect only probable cases. Also, only a few studies support remote diagnosis, but the accuracies attained were around 90%. Few studies use complex models that are not suitable for carrying out diagnoses in fog computing environments. The need for a lightweight model for a fog computing environment that supports remote diagnosis and dual-level diagnosis of dengue to find the confirmed dengue cases has been identified. Ali Mahmoud Ali et al.^25^ proposed improved feature selection using cat swarm optimisation along with K-means and SVM for classification. By improving the feature selection using the optimisation technique, the accuracy increased from 81 to 100%. Sadia Sabrina Prome et al.^26^ has predicted dengue cases in 11 districts using a dataset from Bangladesh’s weather forecast. The best results were obtained by the supper vector regression model with optimised hyperparameters, which showed the lowest mean absolute error of 4.112. In this work, seven machine learning algorithms, including three ensemble learning techniques, were used to forecast dengue cases. Xinyi Pu et al.^8^ have found new diagnostic targets for the diagnosis of Tuberculosis. Peripheral blood was sequenced to screen the diagnostic targets. A novel hybrid directed hypergraph convolution network (H-DHGCN) has been proposed by Qiongjie Cui et al.^27^ to model the high-order relationships of the human skeleton with directionality. Yanfei Jia et al.^28^ have addressed the structural distortions while resolving the retinal fundus utilising a pre-trained U-Net model to create a structural segmentation map of the retinal vasculature utilising the segmentation map as the structural prior. Anas Bilal et al.^29^ have proposed a hybrid CNN-SVD (Convolutional Neural Network-Singular Value Decomposition) model with data pre-processing and feature extraction for detecting vision threatening diabetic retinopathy. Anas Bilal et al.^30^ have proposed an enhanced Quantum-Inspired Binary Grey Wolf Optimizer (EQI-BGWO) with a self-attention capsule network for the detection and classification of vision threatening diabetic retinopathy (VTDR). Anas Bilal et al.^31^ have proposed a novel AI-driven vision threatening diabetic retinopathy (VTDR) detection method that combines various models via majority voting. A hybrid convolutional neural network-singular value decomposition (CNN-SVD) model is used for feature extraction, pre-processing, data augmentation, and classification using an upgraded support vector machine with radial basis function (SVM-RBF) in conjunction with a decision tree (DT) and K-nearest neighbour (KNN). The proposed model has been validated on the IDRiD dataset, which showed an accuracy of 99.89%. Anas Bilal et al.^32^ have proposed a novel two-stage Diabetic Retinopathy (DR) detection method comprising of Optic Disk (OD) and Blood Vessels (BV) segmentation, as well as DR classification using transfer learning. Anas Bilal et al.^33^ have suggested an approach which involves preprocessing, feature extraction and classification. Classifiers used in the classification step include support vector machines (SVM), K-nearest neighbours (KNN), and binary trees (BT). To perform this investigation, several severity of disease grading databases were used. Xiang Feng et al.^34^ have proposed a new technique, which is the fusion of a graph neural network and a multi-layer perceptron for data analysis of single-cell RNA-seq. Anas Bilal et al.^35^ have focused on developing sophisticated in-silico approaches with ensemble learning methodologies to identify the modified 5-methylcytosine (m5c) sites in RNA. The encoded data was processed using ensemble models, which included bagging and boosting approaches. These models were then carefully validated using independent testing and 10-fold cross-validation. Figure 1 gives an overview of different layers used in smart healthcare^36^. Input data is received from the edge layer. Those data is sent to the fog layer or directly to the cloud based on the situation. As the fog layer is closer to the edge layer, local processing that needs immediate action is carried out swiftly^37^. Other important health information that needs to be stored for future analysis or knowledge mining is stored in the cloud layer. During an outbreak of disease, it is important to take speedy action. So in that case, the fog layer helps in sending the diagnostic information much faster than processing it in the cloud, which is several kilometers away. Together with the fog layer, the fog servers are dispersed geographically and carry out calculations near endpoints. At the network edge, each fog server has the capacity to handle large amounts of data. As a result, very little burden is moved to the cloud for processing and archiving. This makes it possible to connect a large number of endpoints to the central cloud, which produces a substantial volume of data^36–38^.

**Fig. 2:**
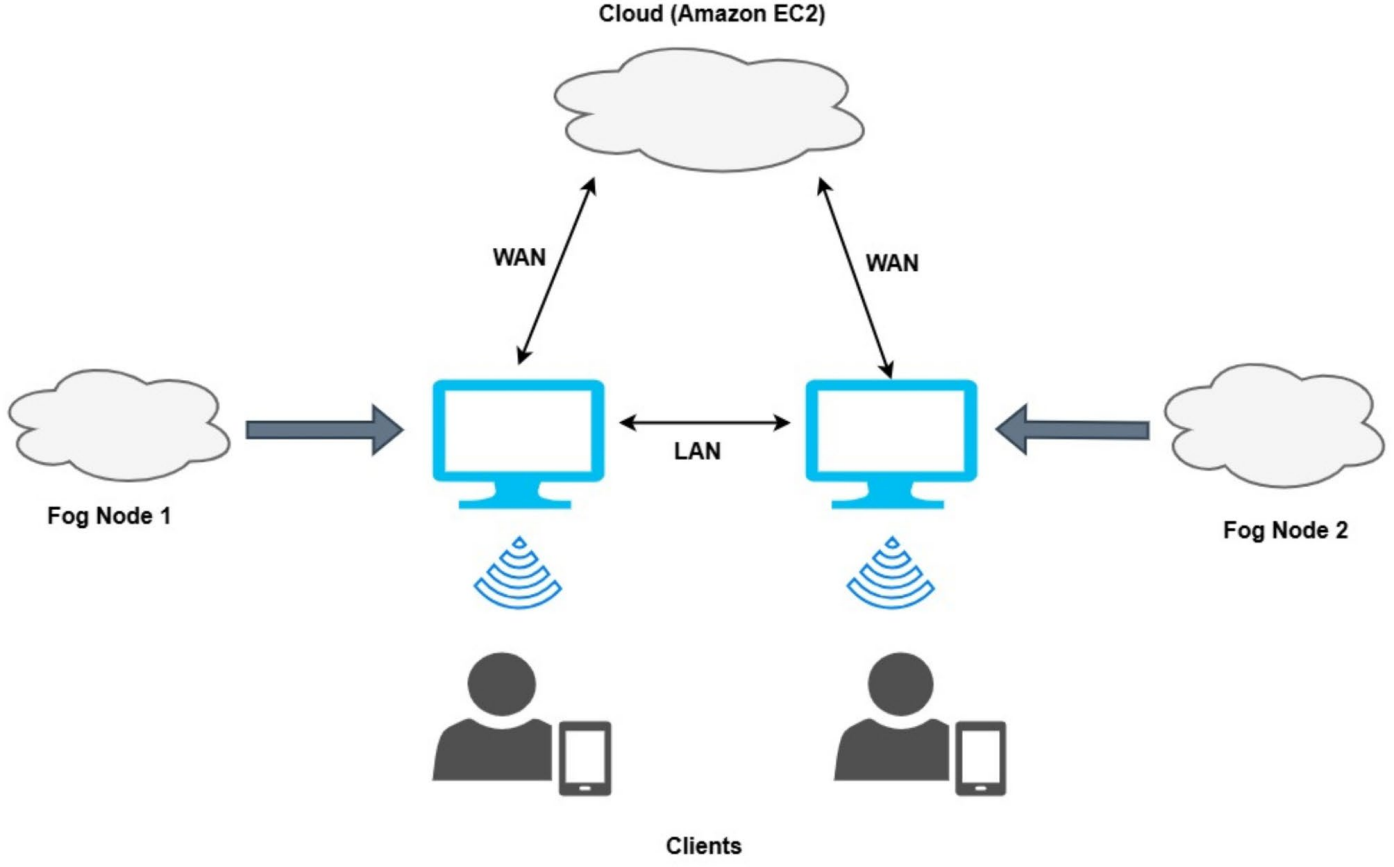
Basic Fog Computing Framework.

According to Fig. 2, after receiving the patient’s data, the edge devices forward it to the appropriate fog node for processing. The fog node is where the data processing and segregation occur, and the fog stores the information locally. Only data that has been permanently stored is transferred to the cloud. The routers are linked to the Amazon EC2 Cloud via Wide Area Network (WAN) and to one another via Local Area Network (LAN). The routers are also equipped with the “Wireless Access Point” (WAP) feature. This enables mobile and smart device access to the fog nodes as well as the Amazon EC2 Cloud.

## Methods

**Fig. 3:**
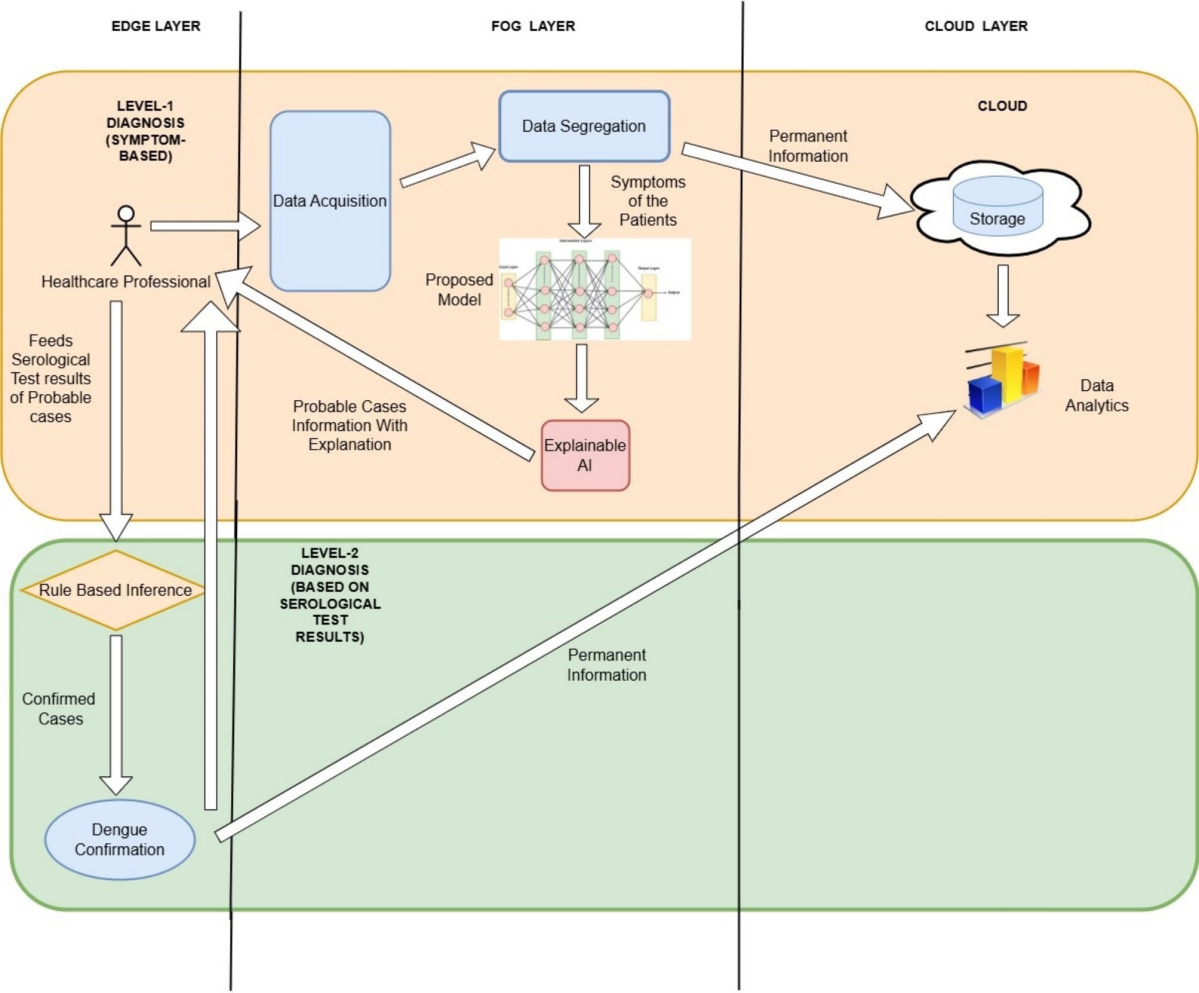
Fog Computing Framework for Dual-Level Diagnosis of Dengue.

**Fig. 4:**
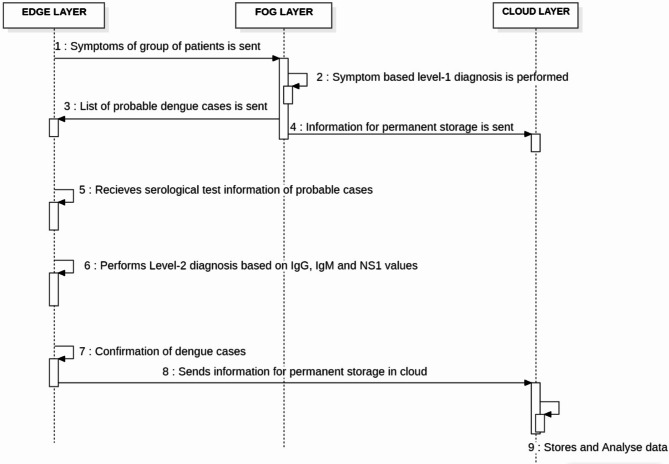
Sequence Diagram explaining the action sequence in the proposed Dual-Level Dengue Diagnosis Framework.

Dengue has a number of disabling symptoms that are comparable to those of other illnesses, although it is not always fatal. People who have dengue usually have a high fever, severe body ache, nausea, appetite loss, and different kinds of skin rashes. Patients frequently endure a dramatic decline in health, even if they do not exhibit any particular symptoms in the first two weeks after infection^39^. The diagnosis of dengue comprises two levels which includes diagnosis based on the symptoms and then the serological tests which include antigen and antibodies detection^4,40^. Antigen Detection: Immunochromatographic or immunofluorescence studies on serum can identify the NS1 antigen, which is essential for DENV replication. Antibody Detection: ELISA is used to detect IgG and IgM in serum and cerebrospinal fluid^2,40^. In symptomatic diagnosis, only the probable cases are identified. The only way to confirm dengue cases is through serological tests. As wrong treatments are started with the symptomatic diagnosis, it is ideal to have both steps and then proceed with the treatment. Fog computing framework for dual diagnosis of dengue has been given in Fig. 3 and the corresponding sequence diagram explaining the sequence of actions in the diagnostic framework of dengue is shown in Fig. 4. The proposed framework for dengue has edge, fog and cloud layers. Symptoms of a group of patients are transmitted for processing from the edge layer to the fog layer. The proposed MLP (Multi-layer Perceptron) model resides in the fog layer and helps in the diagnosis of probable cases of dengue based on the symptoms. The list of probable cases is then sent from the fog layer to the edge. The cloud receives additional data for long-term storage. In the edge layer, the serological test information of probable cases is given as input. Then, by using rule-based classification (Level-2 Diagnosis), dengue cases are confirmed. Information is stored in the cloud permanently for analysis. Healthcare professionals are responsible for giving input to each level of diagnosis. The result from the level-1 diagnosis influences the inputs given by the healthcare professional for the level-2 diagnosis.

### Workflow and dataflow in the proposed dual-level dengue diagnosis framework

In the level-1 diagnosis, the symptoms of the patients are sent collectively to the fog layer along with the patient details. In the fog layer, the data acquisition module receives the data and forwards it to the data segregation module. It segregates patient information from the disease-related information. The data related to the symptoms are sent to the proposed model and permanent information collected is sent to the cloud for storage. The predictions are later explained using explainable AI. Information about the probable cases along with the explanation is sent to the healthcare professional from the fog layer to the edge layer.

The healthcare professional then takes the serological test results of the probable cases and passes that to the level-2 phase of diagnosis. In Level-2 diagnosis, the serological test results of the probable cases are used to find the confirmed cases using the rule-based inference method in the edge layer itself. Later, the information about the confirmed cases is sent to the cloud for permanent storage.

## Steps in level-1 diagnosis of dengue based on symptoms

Symptoms of the patients are used to make a level-1 diagnosis of dengue. The fog layer which contains the suggested model, is where the diagnosis is made. The dataset used to train and test the model, various techniques, and different models that were compared to the proposed models have all been covered in this part.

### Dataset used

The dataset has been obtained from a publication^41^ in the journal JMIR Mhealth Uhealth under the National Library of Medicine which is part of the National Institute of Health (NIH). Real-time data was collected during the study. There are a total of 18 columns and 177 entries in the dataset. Detailed information about the dataset is given below in Table 1. In Table 1,0 to 17 denotes the column. 0 is the target variable and 1 to 17 are independent variables in the dataset. The value of the variables is either 0 or 1 which is held by Value column. It also includes Non-Null count and Dtype which describe the count of not null entries for each feature and datatype respectively.

**Table 1:** Description of the dataset.

	Column	Value	Non-Null Count	Dtype
0	Comfirmed DF	0/1	177	int64
1	DF ever before	0/1	177	int64
2	Family DF history	0/1	177	int64
3	A personal history of mosquito bites within the previous 1 week	0/1	177	int64
4	A high fever ( > = 19 °C)	0/1	177	int64
5	Biphasic fever	0/1	177	int64
6	Erythema	0/1	176	int64
7	Skin rash	0/1	177	int64
8	Petechia	0/1	177	int64
9	Headache	0/1	177	int64
10	Myalgia	0/1	177	int64
11	Abdominal pain	0/1	177	int64
12	Vomiting	0/1	177	int64
13	Soft (watery) stool	0/1	177	int64
14	Cough	0/1	177	int64
15	Sore throat	0/1	177	int64
16	Anorexia	0/1	177	int64
17	Weak sense	0/1	177	int64

### Data preprocessing

Preprocessing is an important step in the workflow of machine learning. It is important for maintaining consistency of the data making it suitable for analysis and modeling. The entire dataset is checked for missing values, incorrect entries, and other inconsistencies. The Erythema column in the dataset had one null entry for which mean imputation has been used to overcome the problem. The null entry has been filled by mean of the column.

### Exploratory data analysis and feature reduction

A strong exploratory data analysis (EDA) tool, heat maps help with pre-processing by providing a rapid visual depiction of the patterns, relationships, and problems of the dataset^19^. It was found that the ‘DF ever before’ column had value 0 as the entry for all rows and did not contribute to the performance of the models. Hence that column has been removed to reduce the overall learning and testing time of the models.

#### Pearson correlation co-efficient

After removing ‘DF ever before’, the correlation heat map has been generated for the dataset using the Pearson Correlation method. The degree and direction of the linear relationship between two variables are assessed statistically by the Pearson correlation coefficient (r). Finding the degree to which two variables are linearly connected is a common statistical technique. The formula for finding the Pearson correlation coefficient (r) is given below in Eq. (1).

1 $$\:\:r\:=\:\sum\:\:\left(\begin{array}{cc}{\text{x}}_{\text{i}}\:-&\:\stackrel{-}{x}\end{array}\right)\left(\begin{array}{cc}{y}_{i}-&\:\stackrel{-}{y}\end{array}\right)/\sqrt{\left(\sum\:{\left(\begin{array}{cc}{\text{x}}_{\text{i}}\:-&\:\stackrel{-}{x}\end{array}\right)}^{2}\sum\:{\left(\begin{array}{cc}{y}_{i}-&\:\stackrel{-}{y}\end{array}\right)}^{2}\right)}\:$$

Where x_i_ and $\:{y}_{i}$ denotes individual data points of variables X and Y

$\:\stackrel{-}{x}-\:$mean of the X values

$\:\stackrel{-}{y}-\:$mean of the Y values

$\:\sum\:-\:$Summation across all data points

The range of $\:r$ is from − 1 to + 1

$\:r=\:+1\:$means perfect positive linear relationship (i.e. when X increases, Y also increases proportionally)

$\:r=\:-1\:$means perfect negative linear relationship (i.e. when X increases, Y decreases proportionally)

$\:r=\:0\:$means no linear relationship

**Fig. 5:**
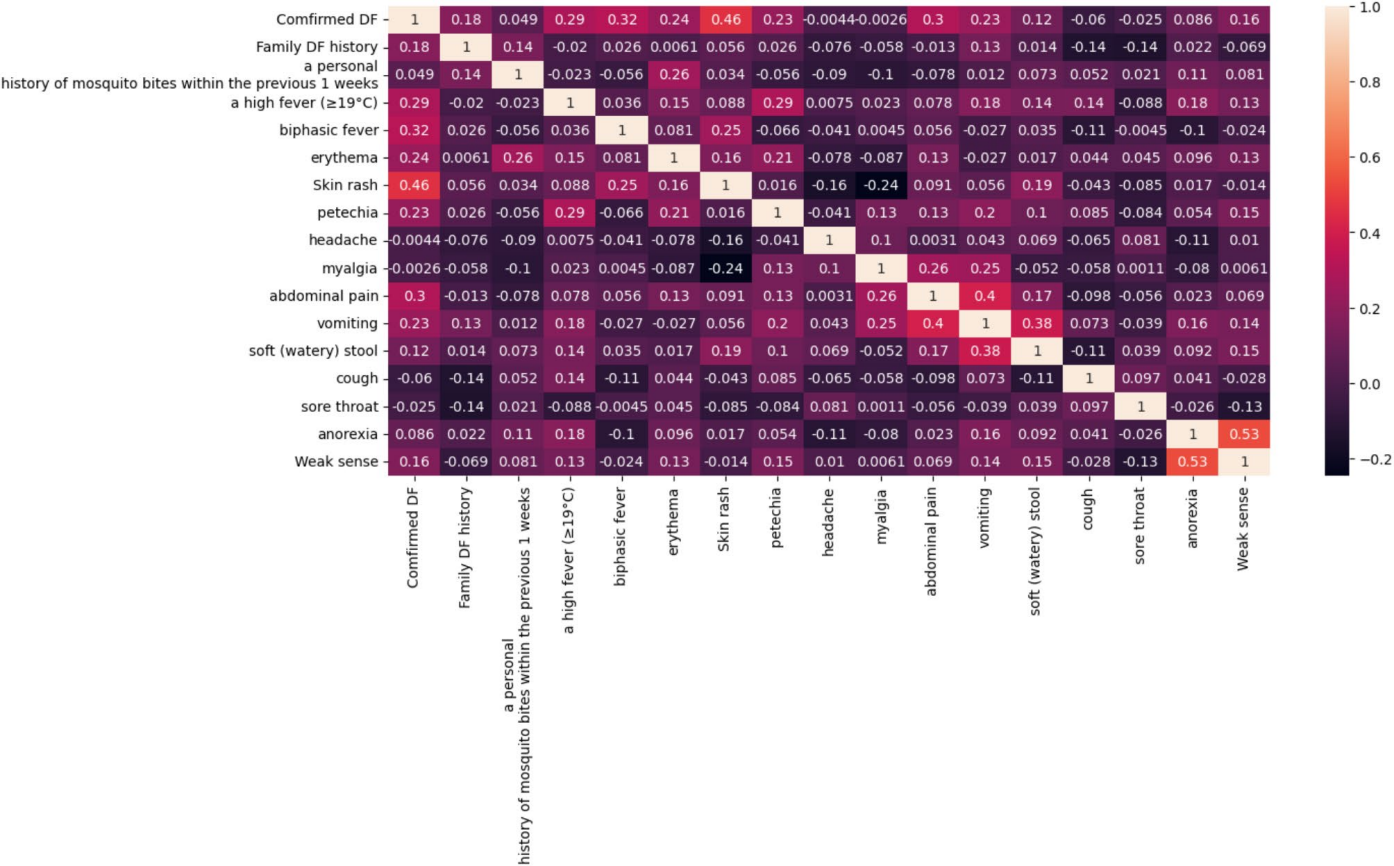
Heat map for the dataset after removing ‘DF ever before’ column.

Figure 5 depicts the heat map where row and column are the features and the entries inside the grid are correlation co-efficient between the two features.

‘Comfirmed DF’ has been selected as the target feature in the dataset. And the column corresponding to ‘Comfirmed DF’ is fetched. It holds the correlation coefficient values between that feature and the remaining independent features. Sorting is done to identify features that are most positively or negatively correlated with the target variable. It helps prioritize the features for further analysis or feature selection^42^. Table 2 holds the correlation coefficient between the target feature ‘Comfirmed DF’ and the other independent features. Some features like Skin rash, biphasic fever, abdominal pain has positive correlation with the target features whereas features like cough, sore throat, and headache have a negative correlation with the target feature. Features like weak sense, vomiting, and Family DF history have a moderate correlation with the target feature.

**Table 2:** Table showing correlation co-efficient between target feature and the independent features.

Cough	− 0.059742
Sore throat	− 0.024655
Headache	− 0.004437
Myalgia	− 0.002583
A personal\nhistory of mosquito bites within the previous 1 weeks	0.049147
Anorexia	0.085678
Soft (watery) stool	0.123303
Weak sense	0.162009
Family DF history	0.178582
Petechia	0.226089
Vomiting	0.232850
Erythema	0.235705
A high fever ( > = 19 °C)	0.286789
Abdominal pain	0.300169
Biphasic fever	0.322055
Skin rash	0.463290
Comfirmed DF	1.000000

The features negatively correlated with the target feature have been removed from the process which includes cough, sore throat, headache and myalgia. Therefore, from a total of 18 features, 1 feature has been considered as a target feature and from the remaining 17 features, 5 features (DF ever before, cough, sore throat, headache, myalgia) are removed. The remaining 12 features are considered as independent features.

### ML and DL models

Following is the list of Machine Learning (ML) / Deep Learning (DL) models analysed in this study using the same dataset. In each case K-fold cross validation with K value as 5 has been carried out.

#### Decision tree

Although decision trees which are supervised learning approaches can be used to tackle both regression and classification problems, they are mainly used to address classification issues^19^. Internal nodes represent the features of a dataset, branches represent the decision rules, and each leaf node represents the outcome in this tree-structured classifier. The divide-and-conquer strategy is used by the decision tree (DT) algorithm^43,44^. DT are of two types - classification trees and regression trees. The two most well-known and often utilized DT algorithms are C4.5 and EC4.5. Decision trees for classification has been used in our study with K-fold cross validation.

#### Random forest

A classifier known as Random Forest uses many decision trees on different subsets of the input dataset and combines the results to increase the expected accuracy of the dataset. As the random forest employs a large number of trees to predict the class of the dataset, it is possible that some decision trees will predict the correct result while others will not^19,39,44–47^.

#### Support vector classifier (SVC)

One kind of machine learning model that can be applied to classification tasks is a support vector classifier. The objective of the support vector classifier is to identify a decision boundary that maximally divides the two classes based on a set of training samples that are each assigned to one of the two classes. Finding the best hyperplane in an N-dimensional space to efficiently divide data points into distinct classes in the feature space is the main goal of the SVC algorithm. The technique makes sure that the distance between the nearest points of various classes are as large as possible.

#### SVC-gridCV

Support Vector Machines (SVM) are the foundation of the machine learning classification model known as SVC (Support Vector Classifier)^25^. GridSearchCV is a scikit-learn technique that searches a given parameter grid to optimize hyperparameters. To get the optimal set of hyperparameters for an SVM classification task, SVC and GridSearchCV are combined.

#### Logistic regression

A statistical method called logistic regression connects an array of independent variables either continuously or discretely to a dependant variable that is binary. It is a strong tool that generates reliable models. It makes dependent data predictions by looking at the association between one or more independent variables that are already existent^39,44–46^.

#### Recurrent neural network (RNN)

Conventional neural networks handle inputs and outputs separately. But in order to make precise predictions, tasks like guessing the next word in a sentence need knowledge about the words that came before it. Recurrent Neural Networks (RNNs) were created to overcome this restriction. In order to retain knowledge from earlier inputs, recurrent neural networks add a method where the output from one stage is given back as input to the next. Because of their nature, RNNs are ideal for tasks like predicting the next word in a phrase, where context from previous steps is crucial^48^.

#### K-nearest neighbour (KNN)

The KNN classification method is a nonparametric approach that was created in 1951 by Evelyn Fix and Joseph Hodges. Regression analysis and classification are two applications of KNN. KNN classification results in class membership. The item is classified using voting processes. The distance between two data samples is calculated using Euclidean distance algorithms^43,45–47^. KNN classification has also been used in our study.

#### Extreme gradient boosting (XGBoost)

The XGBoost algorithm stands for Extreme Gradient Boosting (XGBoost). Among all machine learning algorithms, it is highly regarded due to its speed and performance. Another noteworthy aspect of it is its portability, which allows it to run on any platform and integrate with a variety of existing systems. Because it adheres to the parallelization idea, it typically performs faster than the other boosting methods^19,39^.

#### ExtraTrees

A type of ensemble learning technique called the Extremely Randomised Trees Classifier, or Extra Trees Classifier, creates its classification result by merging the output of several de-correlated decision trees collected in a “forest.” With the exception of how the decision trees of the forest are constructed, it is conceptually the same as a Random Forest Classifier. The original training sample is used to build each Decision Tree in the Extra Trees Forest. The decision tree must then select the optimal feature to split the data based on a statistical criterion (often the Gini Index) from a random selection of k features from the feature-set at each test node^19,44^.

#### Deep neural network (DNN)

A DNN differs from a neural network (NN) only in that it contains multiple hidden layers between the input and output layers. Deep learning-based methods work particularly well with large datasets^49^. Large deep neural networks typically require high-performance computing environments for training and development, in contrast to shallow neural networks. The task at hand determines the amount of resources needed.

#### AdaBoost

The ensemble learning method known as Adaptive Boosting (AdaBoost) was initially created to improve the efficiency of binary classifiers. Boosting is accomplished in AdaBoost by joining a number of weak classifiers. Every weak classifier makes an effort to fix samples that the weak classifier misclassified^39,46^.

#### NaiveBayes

Based on Bayes’ theorem, the naive Bayes method assumes that each feature in a dataset is conditionally independent of the output class. The probability of a hypothesis is expressed by the Gaussian Naive Bayes method. This technique works best with continuous data, but it also works incredibly well with classifying data. This model is straightforward but efficient^39,45,46^.

### Proposed grid search optimized lightweight MLP model with batch normalisation

**Fig. 6:**
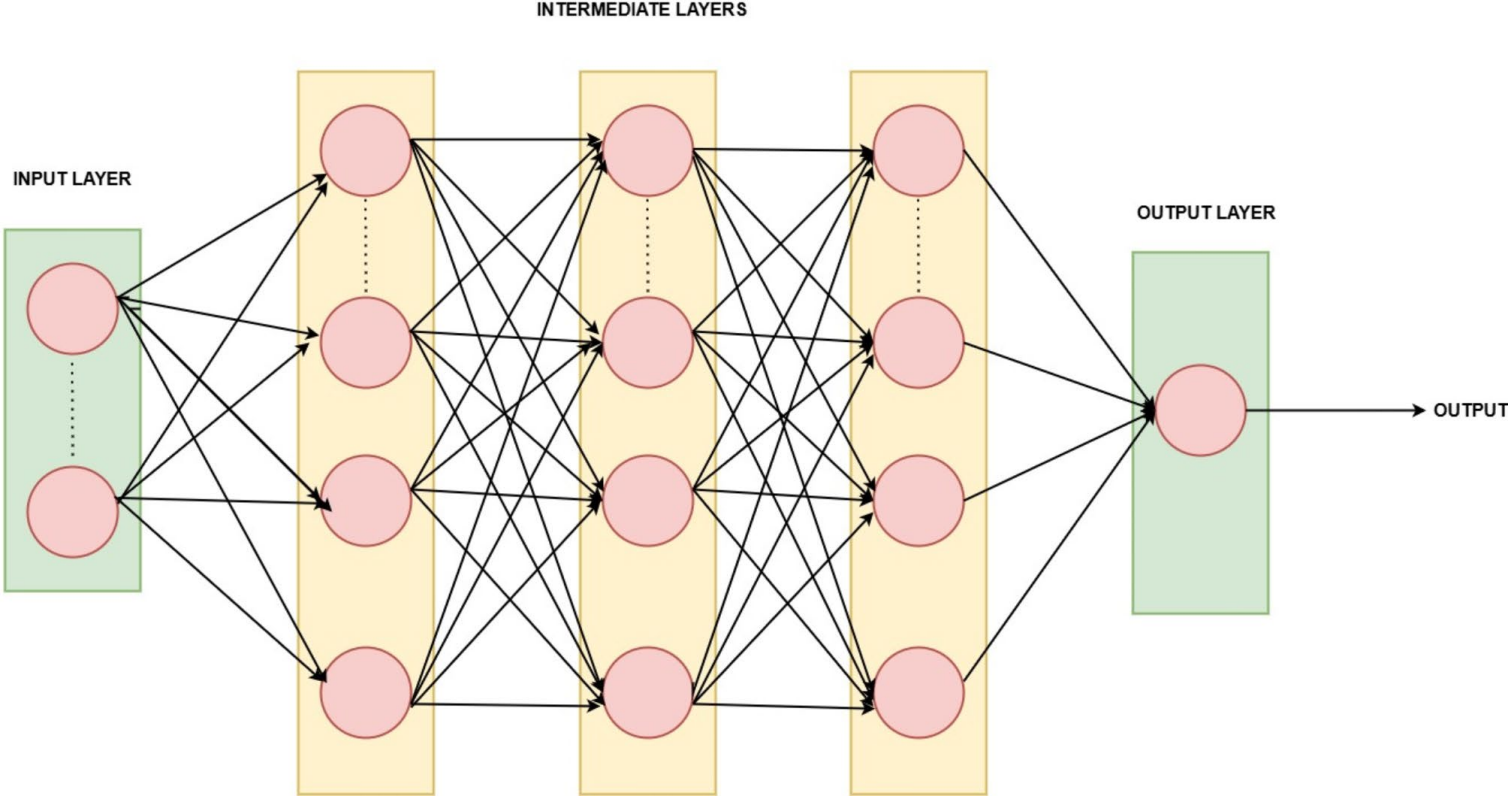
Proposed Lightweight MLP with three hidden layers.

An artificial neural network with many node levels, including input, output, and one or more thick layers, is called a multilayer perceptron (MLP). It is a strong model that can handle both organized and unstructured data, and it can recognize intricate patterns in the data^39^. In the proposed lightweight MLP model shown above in Fig. 6, there are four dense layers. Among the four, three layers are hidden and one is the output layer. The three intermediate or hidden layers are made up of ReLU activation function whereas sigmoid activation function is used for the output layer to perform binary classification.

#### ReLU and sigmoid activation function

ReLU(Rectified Linear activation function) is defined mathematically by Eq. (2) given below,

2 $$\:f\left(x\right)=\left\{\begin{array}{c}x,\:\:x>0\\\:0,\:\:x\le\:0\end{array}\right.$$

where if *x* is positive, the output is *x*. And if *x* is 0 or negative, the output is 0. The ReLU function “clips” all negative inputs to zero, introducing non-linearity even though it appears linear for positive values. Neural networks have the capacity to learn intricate patterns because of this non-linearity. In neural networks, the sigmoid activation function is frequently used, especially in the output layer for tasks involving binary classification. Another name for it is the logistic function. The sigmoid function is defined by the formula in Eqs. (3),

3 $$\:f\left(x\right)=\:\frac{1}{1+{e}^{-x}}$$

Where x is the input to the sigmoid function and the output of the function ranges between 0 and 1. Wherever normalized probabilities are required, such as in binary classifiers or binary decision-making systems, sigmoid is effective. Sigmoid function gives a S – shaped curve. So it is called as Sigmoid function. The curve asymptotes to 1 for positive inputs, 0 for negative inputs and 0.5 for inputs around 0. To avoid overfitting, dropout randomly changes a portion of input units to zero during training.

#### Batch normalization

Batch normalization improves generalization and speeds up convergence by normalizing activations. During training, it normalizes the inputs to a layer for every mini-batch, which helps with problems like internal covariate shift and speeds up the rate of convergence of the model. During training, it normalises the inputs so that each mini-batch has a mean of 0 and a standard deviation of 1. Training is stabilized and accelerated by this standardization. In batch normalization, computation of the mean and variance of the mini-batch is the first step, the inputs are then normalized using the mean and variance values which is followed by scaling and shifting of the normalized value. It stabilizes learning by allowing a higher learning rate. As batch normalization is based on mini-batches, it introduces noise into the data. This lessens overfitting by acting as a regularizer.

#### Drop out

Neural networks employ dropout as a regularization approach to avoid overfitting. Each training phase involves a random “dropping out” (i.e., setting to zero) of a portion of the neurons. Because it can no longer depend on certain neurons being present all the time, the model is forced to learn more robust and generalised features. The drop out works in two ways. During training, dropout deactivates neurons randomly. This is mathematically shown as in Eqs. (4),

4 $${\text{Output}}_{i} = \left\{ {\begin{array}{*{20}c} {0,} & {with\:probability\:p} \\ {{\text{Activation}}_{i} ,} & {with\:probability\:1 - p} \\ \end{array} } \right.$$

During testing, the dropouts are not applied. Instead the activations are scaled by (1 − p) to preserve the same anticipated output.

#### Adam optimiser

One of the most used optimization techniques in deep learning is the Adam optimizer. It combines the benefits of two other well-known optimization techniques: momentum (using previous gradients to smooth updates) and AdaGrad (adaptive learning rates). Adam, which stands for Adaptive moment estimation estimates the first moment (mean) and second moment (variance) of the gradients in order to calculate adaptive learning rates for each parameter. A flexible, effective and adaptable optimization technique, the Adam optimizer performs well on a broad range of neural network architectures and datasets.

#### Binary cross-entropy

For binary classification problems, binary cross-entropy quantifies the discrepancy between the true label distribution and the projected probability distribution. It assesses how closely the actual labels match the expected probability of the model. Here in our proposed model, the loss function is employed. Its objective is to determine how well the expected probability matches the actual binary labels (0 or 1).

#### GridSearchCV for hyperparameter tuning

GridSearchCV is a machine learning method used to methodically look for the ideal set of hyperparameters for a particular model. It uses cross-validation to assess the model for every combination after conducting an exhaustive search across a predetermined grid of hyperparameters. GridSearchCV is a robust and systematic approach for hyperparameter optimization of the model. The three main steps carried out in GridSearchCV are hyperparameter tuning, cross-validation and exhaustive search (i.e., all possible hyperparameter combinations in the grid are tested)^19^. For vast search spaces, it can be computationally difficult, but it guarantees the best parameters are identified. Alternatives like RandomizedSearchCV or Bayesian optimization may be more appropriate for larger or more complicated tasks.

#### K-fold cross validation

K-Fold cross validation divides the dataset into K subsets (folds) to evaluate the performance of the model. It lowers the chance of overfitting by assessing the model’s capacity to generalize to a different dataset. It iterates K times and for each iteration-.

It splits the dataset into K fold, use one fold for testing and remaining k-1 folds for training. To obtain an overall estimate, average the performance metric across all folds after K iterations. Here, 5-fold cross validation has been carried out during modeling.

#### Dense layers and output layer in the proposed lightweight MLP model

The proposed lightweight MLP model has three intermediate fully connected dense layers and an output dense layer that is also completely integrated with the sigmoid activation function for tasks involving binary classification. Below is the description of the different layers in the proposed model.

- Dense Layer 1: The first dense layer has the parameter input_shape that defines the input layer. It is a dense layer with ‘unit1’ neurons. We use GridSearchCV and the variable ‘unit1’ is replaced by the values in the hyperparameter grid. Through ReLU activation, the input from all features is passed.
- Dense Layer 2: It is a dense layer with ‘unit2’ neurons where the variable ‘unit2’ is replaced by the values in the hyperparameter grid during GridSearch. Input is received from neurons of the previous layer. ReLU is the activation function used in this layer.
- Dense Layer 3: It is a dense layer with ‘unit3’ neurons where the variable ‘unit3’ is replaced by the values in the hyperparameter grid during GridSearch. Input is received from neurons of the previous layer. ReLU is the activation function used in this layer.
- Output Layer: The output layer is a fully connected layer with 1 neuron and a sigmoid activation function for binary classification.

Dense Layer 1 is followed by a dropout function with a dropout rate 0.3. Also, batch normalisation is carried out after the dropout. Whereas only the dropout function is carried out after dense layers 2 and 3. After the output operation in the output layer, the Adam optimiser is used for optimisation and the loss function that is employed is binary cross-entropy. Binary cross-entropy assesses how closely the actual labels match the expected probability of the model. The model performed efficiently and gave an accuracy of 92% with the following parameters (batch size = 16, dropout_rate = 0.4, unit1 = 64, unit2 = 32, unit3 = 16) after hyperparameter tuning done by the GridSearchCV. Here unit1, unit2 and unit3 specify the number of neurons in each dense layer. The total number of trainable parameters is relatively low compared to deeper or wider networks. Hence it is a lightweight model. The K-fold with n_splits value as 5 has been used in the process. The total parameters used in the model after counting it layer by layer is 3,713. In which there are 3,585 trainable parameters and 128 non-trainable parameters. Model size is 36.35KB approximately. The model size is approximately 14.50 KB. The training time taken per epoch is 0.8 s for running on normal CPU (Central Processing Unit). Total training time is 3 min in CPU.

### Performance evaluation parameters

Accuracy, precision, recall, F1-score and specificity are used to evaluate and compare the performance of the proposed approach.

- True Positives (TP): Both the target class and the classifier predict that it will be positive.
- True Negatives (TN): The target class is likewise negative, as predicted by the classifier.
- False Positives (FP): When the target class is negative but the classifier predicts it to be positive.
- False Negatives (FN): When the target class is positive but the classifier predicts it to be negative.

#### Accuracy

A performance metric that can be used to determine the percentage of correctly classified predictions is accuracy^45,46^. It is expressed using Eq. (5).

5 $$\text{A}\text{c}\text{c}\text{u}\text{r}\text{a}\text{c}\text{y}\:=\frac{\text{N}\text{u}\text{m}\text{b}\text{e}\text{r}\:\text{o}\text{f}\:\text{c}\text{o}\text{r}\text{r}\text{e}\text{c}\text{t}\:\text{p}\text{r}\text{e}\text{d}\text{i}\text{c}\text{t}\text{i}\text{o}\text{n}\text{s}}{\text{T}\text{o}\text{t}\text{a}\text{l}\:\text{n}\text{u}\text{m}\text{b}\text{e}\text{r}\:\text{o}\text{f}\:\text{p}\text{r}\text{e}\text{d}\text{i}\text{c}\text{t}\text{i}\text{o}\text{n}\text{s}}$$

#### Precision

Precision is the proportion of positive attempts that were properly classified relative to the total number of positive predictions^45,46^. Equation (6) can be used to describe precision,

6 $$\:\text{P}\text{r}\text{e}\text{c}\text{i}\text{s}\text{i}\text{o}\text{n}=\:\frac{\text{T}\text{r}\text{u}\text{e}\:\text{P}\text{o}\text{s}\text{i}\text{t}\text{i}\text{v}\text{e}\text{s}\left(\text{T}\text{P}\right)}{\text{T}\text{r}\text{u}\text{e}\:\text{P}\text{o}\text{s}\text{i}\text{t}\text{i}\text{v}\text{e}\text{s}\left(\text{T}\text{P}\right)+\text{F}\text{a}\text{l}\text{s}\text{e}\:\text{P}\text{o}\text{s}\text{i}\text{t}\text{i}\text{v}\text{e}\text{s}\left(\text{F}\text{P}\right)}$$

#### Recall

The ratio of positive predicted outcomes to all predictions in a class is determined using recall^45,46^. Equation (7) can be used to describe the recall,

7 $$\:\text{R}\text{e}\text{c}\text{a}\text{l}\text{l}=\:\frac{\text{T}\text{r}\text{u}\text{e}\:\text{P}\text{o}\text{s}\text{i}\text{t}\text{i}\text{v}\text{e}\text{s}\left(\text{T}\text{P}\right)}{\text{T}\text{r}\text{u}\text{e}\:\text{P}\text{o}\text{s}\text{i}\text{t}\text{i}\text{v}\text{e}\text{s}\left(\text{T}\text{P}\right)+\text{F}\text{a}\text{l}\text{s}\text{e}\:\text{N}\text{e}\text{g}\text{a}\text{t}\text{i}\text{v}\text{e}\text{s}\left(\text{F}\text{N}\right)}$$

#### F1 score

The harmonic mean of precision and recall is the F1 Score. The trade-off between the two measurements is balanced by F1 score^45,46^. Equation (8) can be used to describe the F1 Score,

8 $$\:\text{F}1\:\text{S}\text{c}\text{o}\text{r}\text{e}=2.\frac{\text{P}\text{r}\text{e}\text{c}\text{i}\text{s}\text{i}\text{o}\text{n}.\text{R}\text{e}\text{c}\text{a}\text{l}\text{l}}{\text{P}\text{r}\text{e}\text{c}\text{i}\text{s}\text{i}\text{o}\text{n}+\text{R}\text{e}\text{c}\text{a}\text{l}\text{l}}$$

#### Specificity

The percentage of real negatives among all actual negatives is known as specificity, or true negative rate^45^. Equation (9) can be used to describe the specificity, Eq. (9) can be used to describe the specificity,

9 $$\:\text{S}\text{p}\text{e}\text{c}\text{i}\text{f}\text{i}\text{c}\text{i}\text{t}\text{y}=\:\frac{\text{T}\text{r}\text{u}\text{e}\:\text{P}\text{o}\text{s}\text{i}\text{t}\text{i}\text{v}\text{e}\text{s}\left(\text{T}\text{P}\right)}{\text{T}\text{r}\text{u}\text{e}\:\text{N}\text{e}\text{g}\text{a}\text{t}\text{i}\text{v}\text{e}\text{s}\left(\text{T}\text{N}\right)+\text{F}\text{a}\text{l}\text{s}\text{e}\:\text{P}\text{o}\text{s}\text{i}\text{t}\text{i}\text{v}\text{e}\text{s}\left(\text{F}\text{P}\right)}$$

#### Confusion matrix

A performance evaluation tool that breaks down the expected and actual results of a classification model is called a confusion matrix. It provides a thorough grasp of how well any classification model performs, especially in situations when the consequences of false positives and false negatives vary^45,46^.

#### ReceiverOperatingCharacteristic (ROC-Curve)

**Fig. 7:**
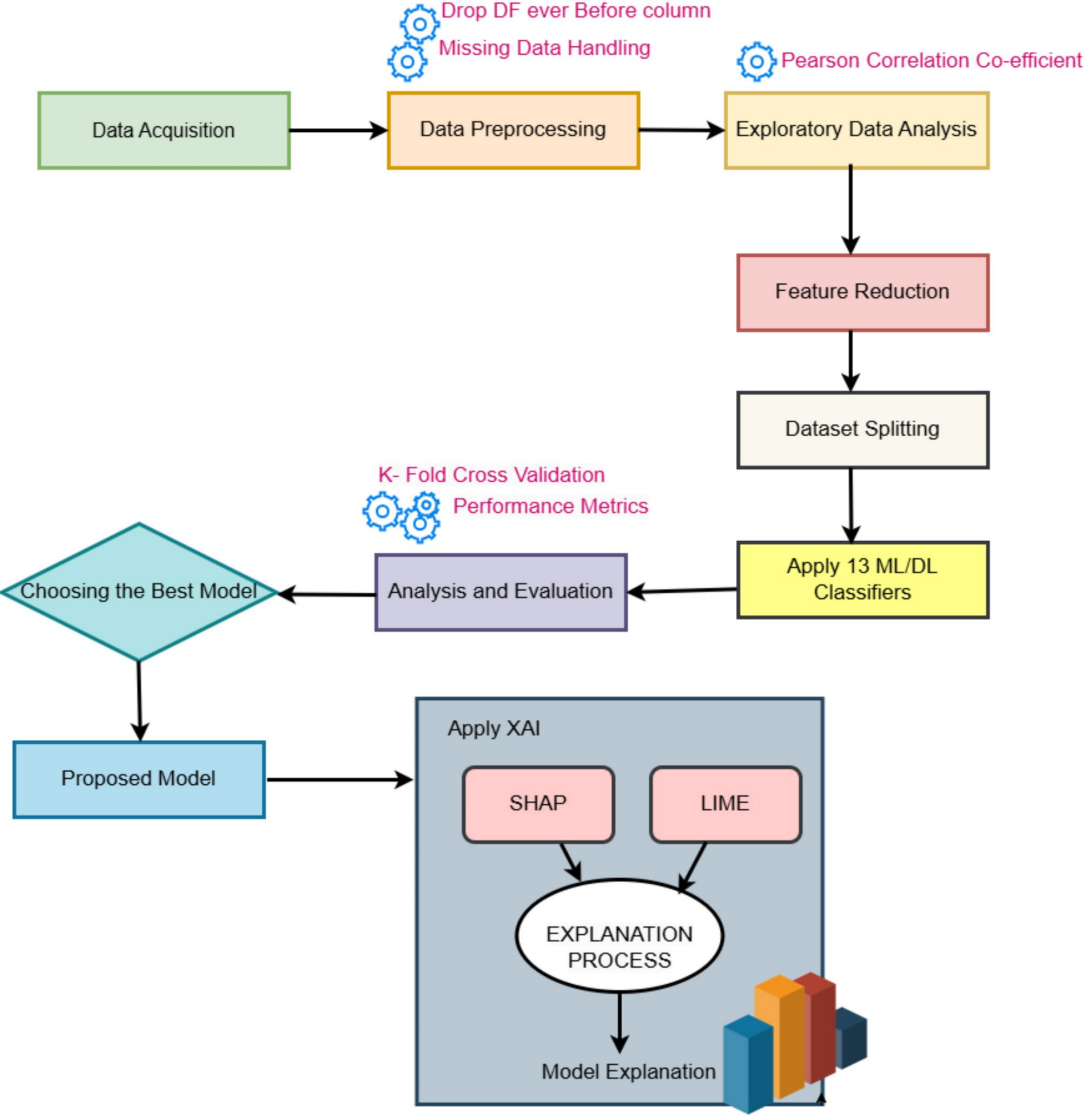
Detailed workflow explaining the steps carried out in Level-1 Diagnosis.

A graphical representation of the capability of a binary classification system when its discrimination threshold is changed is called the Receiver Operating Characteristic (ROC) curve. It is widely used to evaluate how well models that provide probability perform. The X-axis of the curve denotes False Positive Rate (FPR) and the Y-axis denotes True Positive Rate (TPR). A diagonal line accompanies all ROC curves. The model outperforms random guessing when the ROC curve is above this diagonal line. Otherwise, the model performs worse than random and could be improved^46^. The ROC curve for the proposed model is shown in the discussion section. The detailed workflow of the steps in Level-1 Diagnosis is given below in Fig. 7.

## Results

**Fig. 8:**
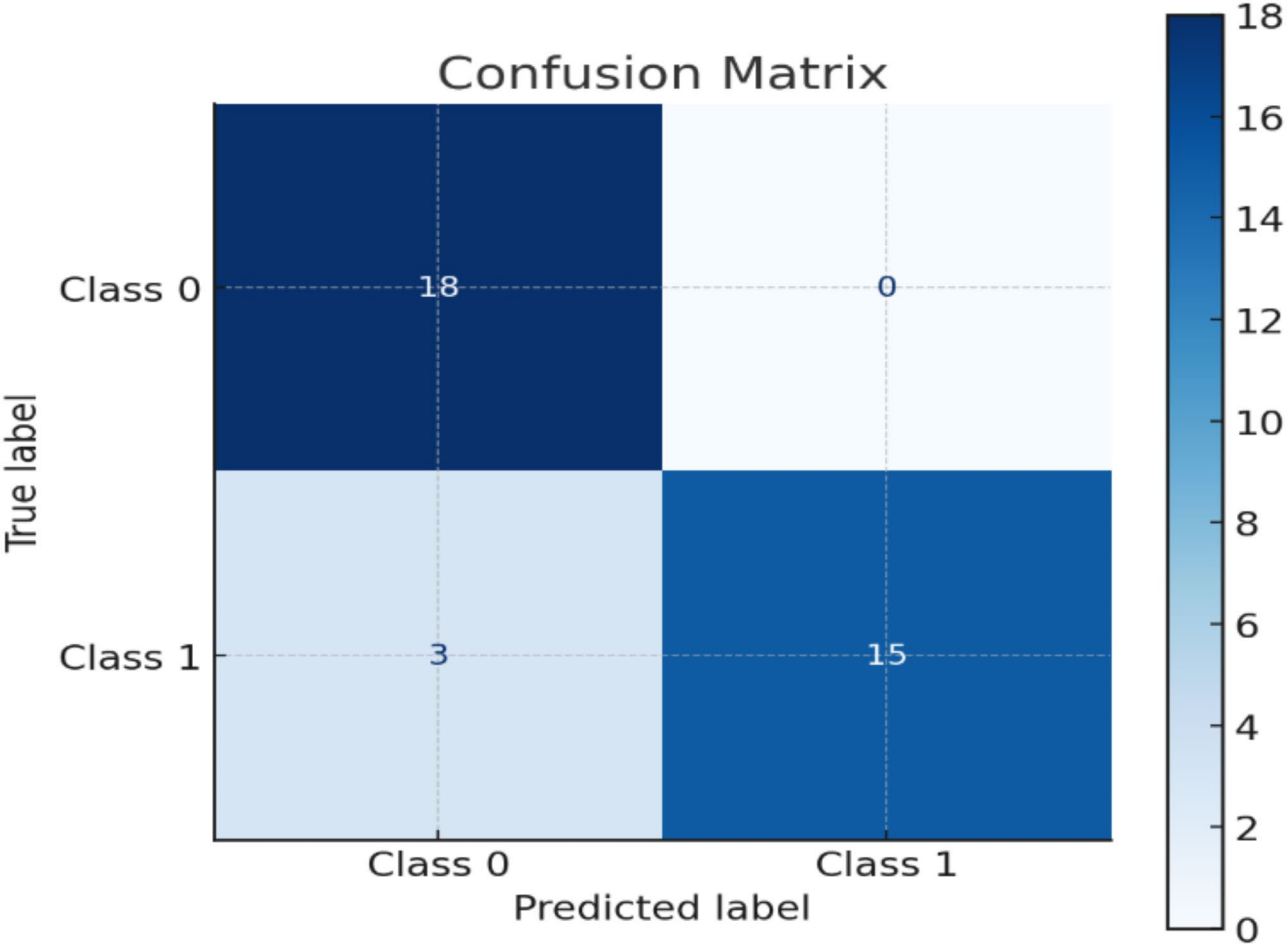
Confusion Matrix for the Performance of the Proposed Model.

Performance metrics of the proposed model have been compared with the performance metrics of other models in Table 3. A detailed analysis has been done, and the basic models have been checked for performance (as shown in Table 3). For all the DL models, the number of epochs set is 200 in common. But the MLP model gave the best results for the problem. MLP was further optimised using grid search, normalised and made light to suit the fog computing environment. Low computational overhead, energy efficiency, reduced memory footprints, low latency for real-time processing, and bandwidth efficiency are some of the advantages of using lightweight MLP in a fog computing environment. Also, the confusion matrix for the proposed model has been shown in Fig. 8. With 100% precision (no false positives), the model accurately predicts every positive result. From the confusion matrix, fifteen ‘class 1’ instances were accurately predicted by the model to be class 1 (True Positive = 15). Eighteen ‘Class 0’ occurrences were accurately predicted to be Class 0 by the model (True Negative = 18). The model predicted 0 instances of Class 1 that were actually Class 0 (False Positives = 0). The model predicted 3 instances of Class 0 that were actually Class 1 (False Negatives = 3).

**Table 3:** Performance comparison between different ML/DL models with the proposed model.

ML and DL Techniques	Accuracy (%)	Precision (%)	Recall (%)	F1-Score (%)	Specificity (%)
Decision Tree	75	71	56	62	78
Random Forest	77	71	56	62	78
SVC	77	80	67	73	83
SVC-gridCV	78	73	61	67	78
Logistic Regression	79	78	65	71	88
RNN	49	53	50	51	48
KNN	77	73	68	70	83
XGBoost	76	77	76	76	83
Extra trees	79	79	79	79	85
DNN	79	79	79	79	83
AdaBoost	85	85	85	85	88
NaiveBayes	67	74	67	63	94
**Proposed optimized Lightweight MLP Model**	**92**	**100**	**83**	**90**	**100**

## Explainablity using SHAP and LIME

Explainable AI (XAI) helps to understand the black box models in ML and reveals the mystery of such models. The degree to which a person could understand the ML choice, explain how the ML model works and address the reasoning behind this decision is known as explainability. Accuracy and explainability are two distinct concerns that should be preserved while creating machine learning models. The interpretability and transparency of machine learning models are critical in the healthcare sector particularly in fields like disease detection (e.g., dengue diagnosis). Decisions of AI systems have a big influence on patient outcomes, and stakeholders such as healthcare professionals, patients, and regulatory bodies require explainability to trust and act on the prediction of the model. This is where Explainable Artificial Intelligence (XAI) technologies such as LIME (Local Interpretable Model-agnostic Explanations) and SHAP (Shapley Additive Explanations) are useful. They aid in clarifying the prediction process of the model which can promote confidence and yield insightful information. Typically algorithms that do well in terms of accuracy provide a clear rationale for their choices and vice versa. The global approach comprehends the overall behavior of the model and how each feature influences the output decision and the local approach clarifies the decision of the model for each instance^50–55^.

### SHAP (Shapley additive explanations)

A strong machine learning technique called SHAP aids in deciphering and elucidating the predictions produced by intricate models^56^. SHapley Additive exPlanations (SHAP) use coalitional game theory to calculate an additive importance score, or Shapley value, for every feature in each individual prediction. These scores are then added up to provide the model’s overall explainability^57^. The advantages that SHAP provides in ML applications are as follows:

1. Increased Transparency: SHAP increases the transparency of black-box models, which promotes stakeholder and user trust. This is particularly important in fields where comprehending model decisions is critical, such as finance, healthcare, and law^56^.
2. Regulatory Compliance: Model judgments must be explicable due to restrictions governing many businesses. SHAP guarantees adherence by offering comprehensible justifications for every choice^56^.
3. Increased user adoption and trust: When end users get the rationale behind a model’s predictions, their likelihood of adopting and trusting the technology is higher. SHAP explanations can make AI-powered apps more user-friendly^56^.
4. Actionable Insights: SHAP offers actionable insights in addition to explanations of predictions. For instance, SHAP can pinpoint important elements for useful characteristics in prediction models, enabling medical professionals to proactively recognize illness^56^.
5. Promotes Collaboration: By bridging the gap between data scientists and non-technical stakeholders, SHAP explanations can promote improved interactions and collaboration. Teams may collaborate more successfully if they have a shared understanding of model behavior^56^.

As MLP is a black box model, the proposed model has been made explainable using SHAP. We have incorporated different techniques like plotting a force plot, dependence plot, summary, and mean plot to reason out the predictions of the model in a better way. This allows the user without any expert knowledge to know the reason behind each prediction, the significance of each feature and how these features relate to one another. Using SHAP, we have produced a summary plot, force plot, mean plot and dependence plot for more explainability of the predictions made by the proposed lightweight MLP model.

#### Summary plot

**Fig. 9:**
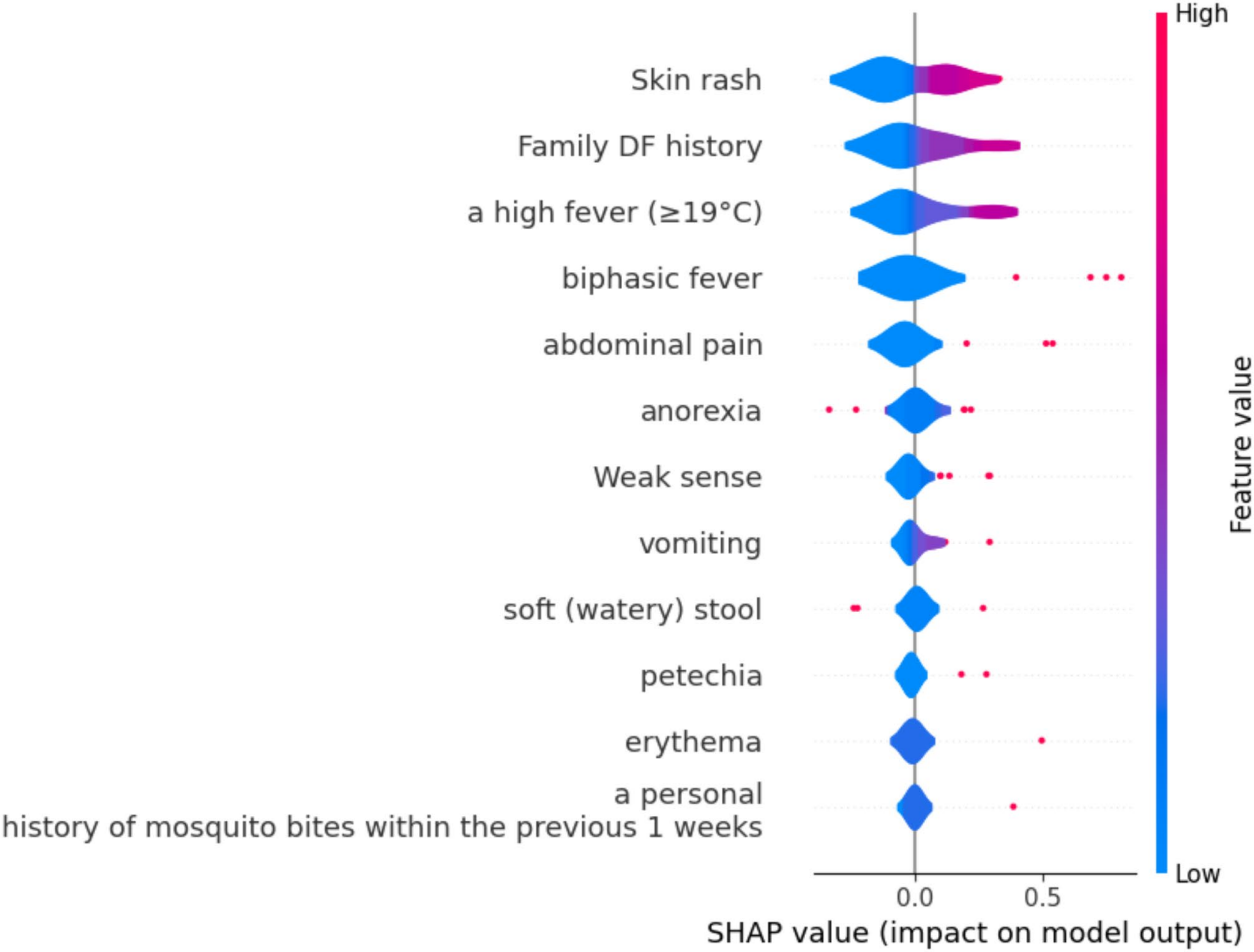
Summary Plot for the Proposed model using S.

This offers information about how each feature contribute to the output of the machine learning model^57–59^. Figure 9 shows the summary plot for the proposed model. The model’s predictions are most affected by features that are higher on the list (such as Skin rash, Family DF history). Higher feature values (shown in red) contribute favorably to the output of the model, while lower feature values (shown in blue) contribute less or negatively. The distribution for “a high fever,” depending on the value of the feature, shows a mixed contribution.

#### Force plot

**Fig. 10:**
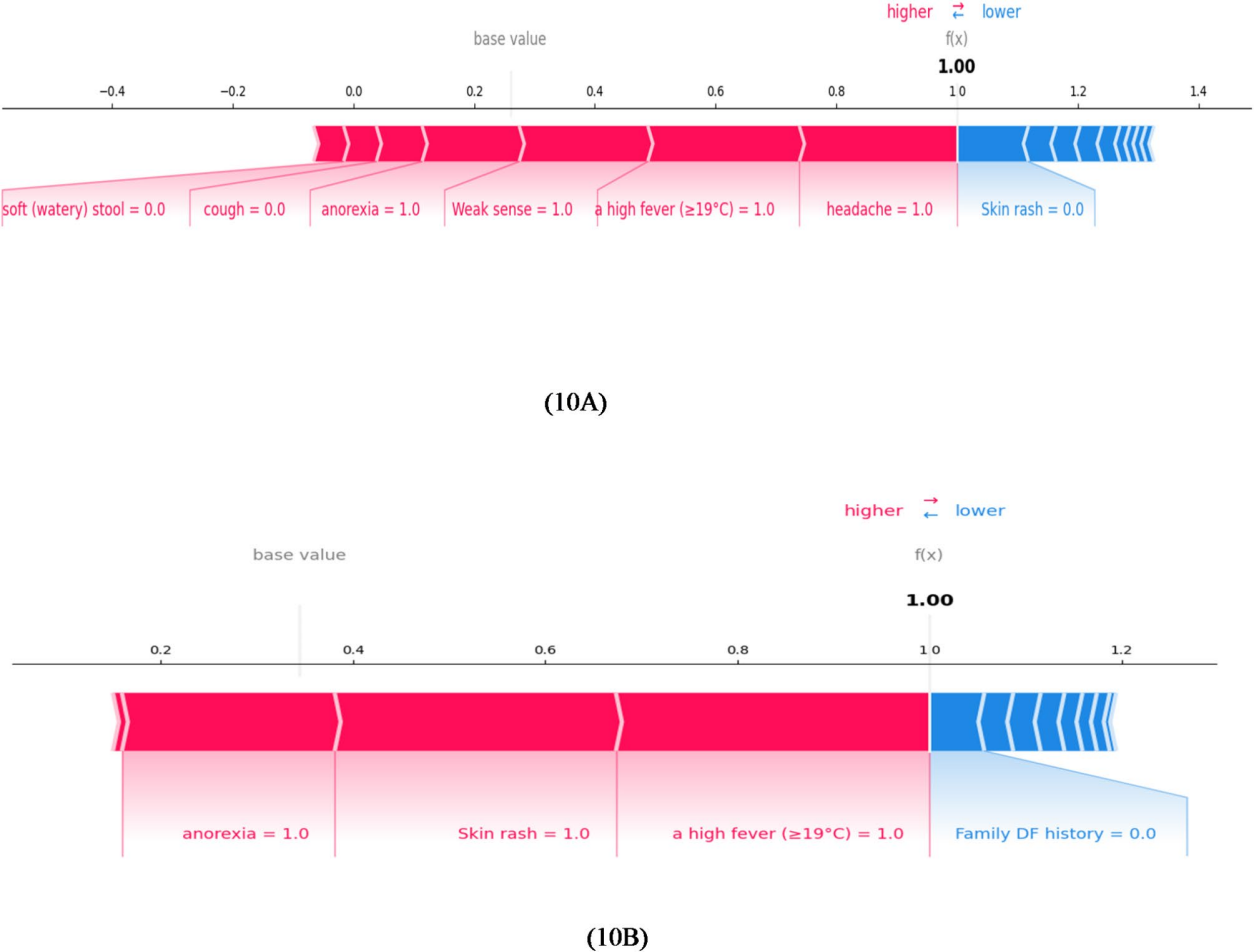
Force Plots explaining the model’s prediction by showing the negative and positive impacts of the features.

A local interpretation of SHAP values with an additive force setup is shown in the plot in Fig. 10. 10 A and 10B are force plots for two different instances from the dataset. This lets the user see the contributions of individual features as the classifier predicts for a single instance (patient), much like the plots described above^59^. The features are divided into red and blue categories based on how much they contribute to increasing or decreasing the score. Nearer the dividing boundary are the features that affect the forecast. Bar size of the feature corresponds to how much of an influence it has on the Light GBM forecast^60^. 10 A, 10B in Fig. 10 are examples of the force plots generated using SHAP. In the case of 10 A, the basic value (around 0.0) was used to start the model. Features such as “headache” and “high fever” pushed the prediction closer to 1.0. On the other hand, although it had less of an impact than other variables, “Skin rash” reduced the forecast. Because “high fever” and other symptoms have such strong positive contributions, the model predicts a high probability (1.0) for the positive class in this scenario. In the case of 10B, the model predicted an extremely confident positive class of 1.0.“Anorexia,” “Skin rash,” and “A high fever” each significantly increased the prediction. “Family DF history” had a minor negative impact on the forecast, but not enough to make a big difference. The model’s prediction of a positive class in this case is explained in detail by these force plots. It is especially helpful in fields where knowing precise contributing features is essential, such as healthcare (e.g., disease diagnosis).

#### Mean plot

**Fig. 11:**
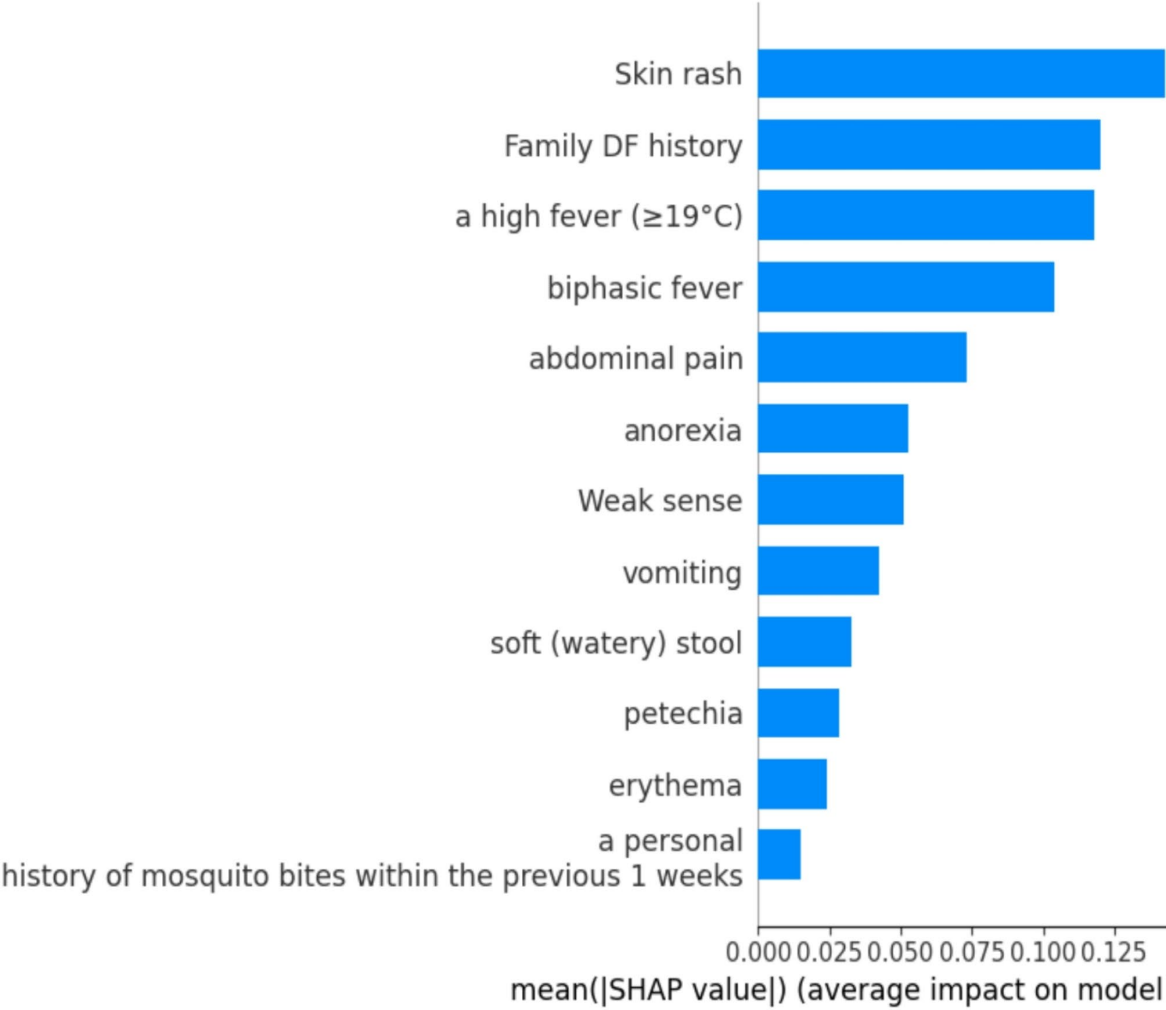
Mean Plot using SHAP.

The global significance of features in a machine learning model based on SHAP values is summarized by a SHAP mean plot also known as a mean absolute SHAP value plot or a SHAP bar plot. It assists us to figure out which features have the greatest overall influence^59^. The mean plot for the features of the model has been shown in Fig. 11.

#### Dependence plot

**Fig. 12:**
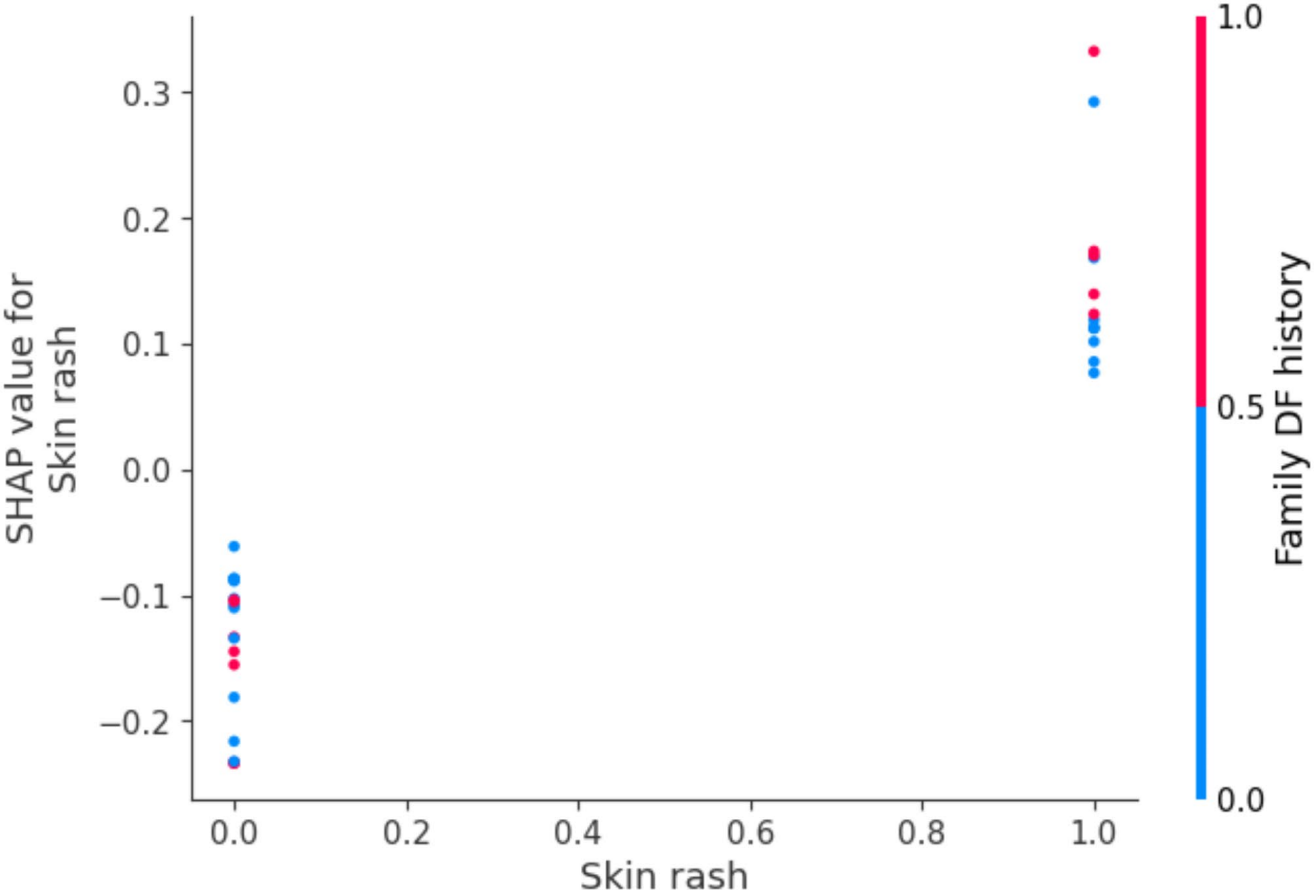
Dependence Plot for the features Skin rash and Family DF history.

A visualization that illustrates the relationship between the values of a particular feature and the corresponding SHAP values is called a SHAP dependence plot. This kind of visualization can show interactions between features and aid in explaining how a feature affects the predictions. It helps in detecting the feature interactions i.e., how one feature impacts another. The Y-axis displays the SHAP value of the feature, while the X-axis displays the feature’s values. The second feature is reflected in the colors^59,60^. Figure 12 shows the relationship between the features ‘Skin rash’ and ‘Family DF history’.

### LIME (Local interpretable model-agnostic explanations)

To interpret the architecture of the suggested model, we used LIME. LIME modifies the input data in order to determine how it affects the predictions of the model. The results of this model-agnostic XAI approach show how each attribute contributed to the prediction.

#### Application of LIME to MLP

By altering the input data and training a basic interpretable model (like linear regression) to roughly represent the MLP’s decision boundary, LIME generates locally interpretable models. LIME repeatedly modifies an input instance and tracks the changes in the prediction of the MLP. To approximate the behavior of the MLP in the vicinity of the instance, a weighted linear model is fitted. This offers local interpretability by emphasising the characteristics that have the most influence on a particular prediction.

#### Prediction probability chart

**Fig. 13:**
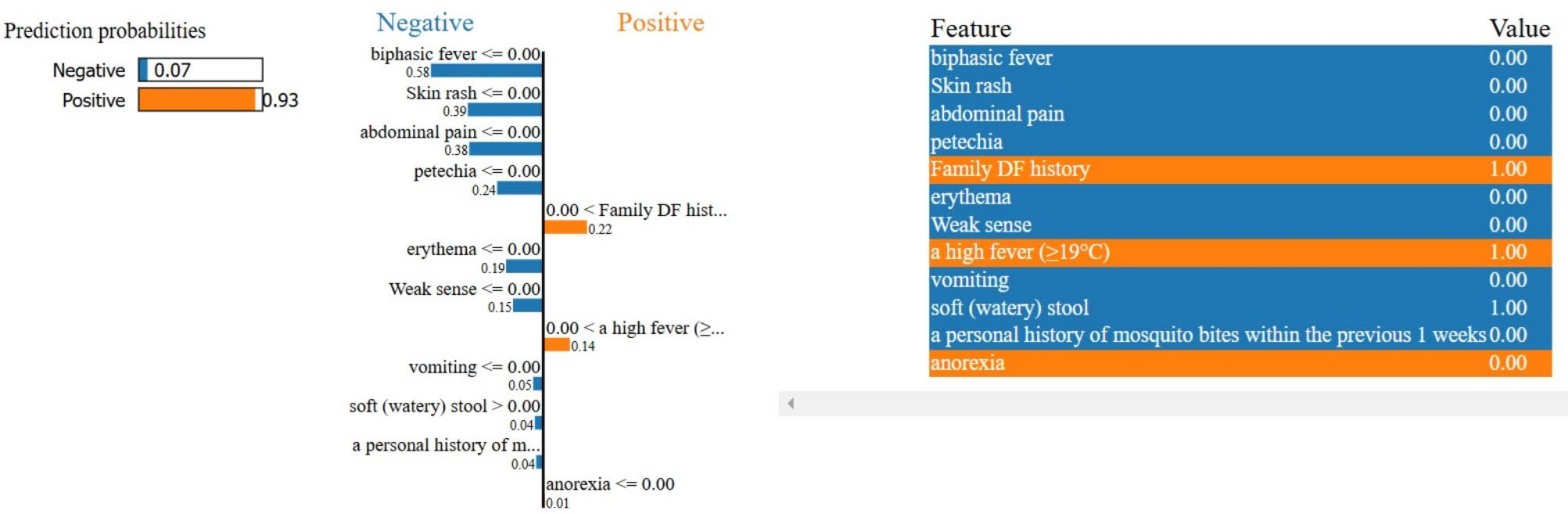
Prediction probability chart given by LIME for an instance predicted by the proposed Model.

For a given instance, a prediction probability chart produced by LIME usually displays the contribution of features to the anticipated likelihood. The features that contribute negatively to classifications are represented by the blue rows, while the parameters with orange rows show a positive impact^60^. The prediction probability chart for a prediction has been shown below in Fig. 13. The features represented in orange contribute positively to the prediction, whereas the features described in blue contribute negatively to the prediction of the model. For the example below, the instance with index 0 was chosen from the dataset.

## Method for level-2 diagnosis (rule-based inference)

**Fig. 14:**
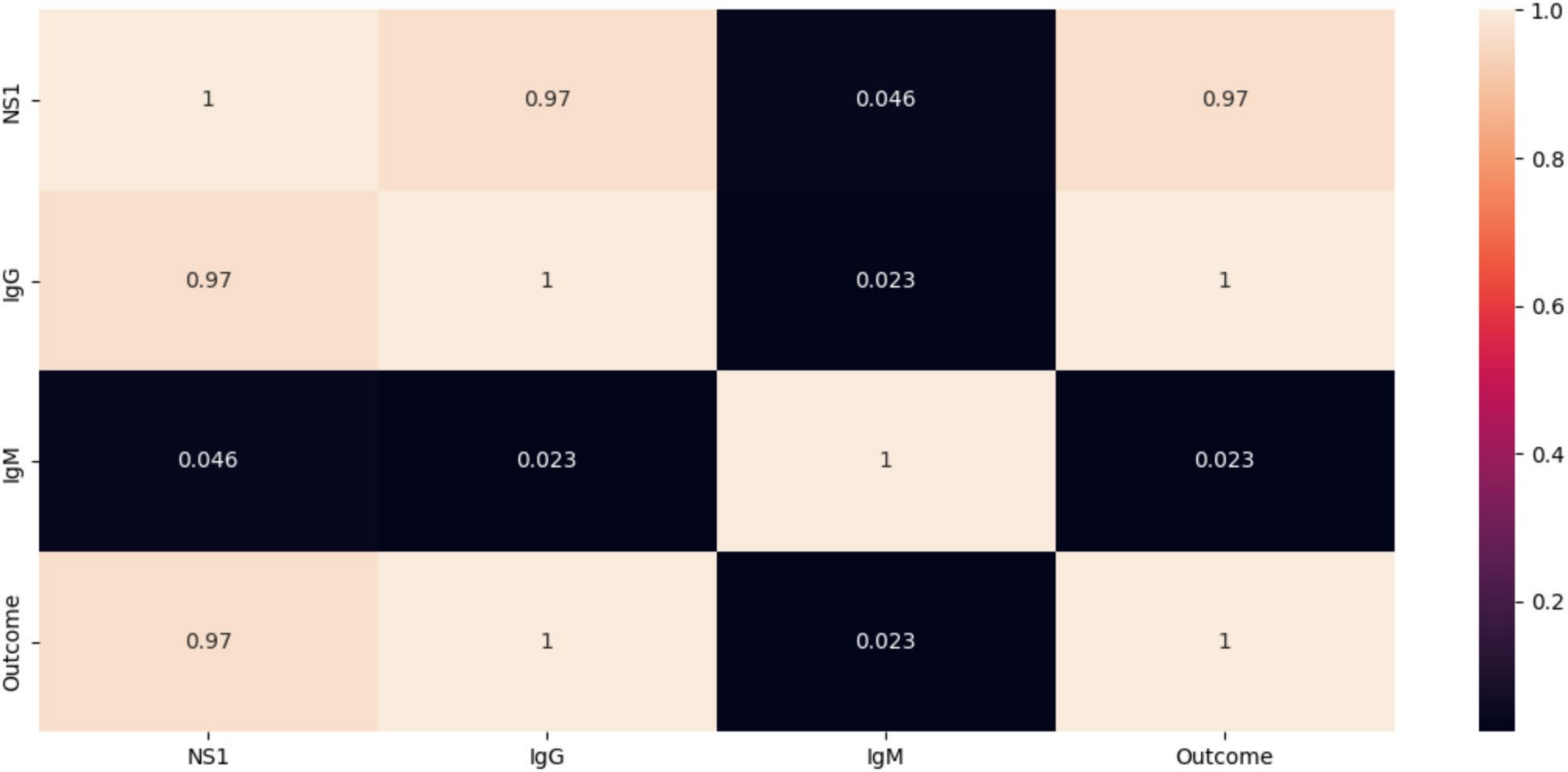
Heatmap for the Reduced Features in the Dataset.

**Fig. 15:**
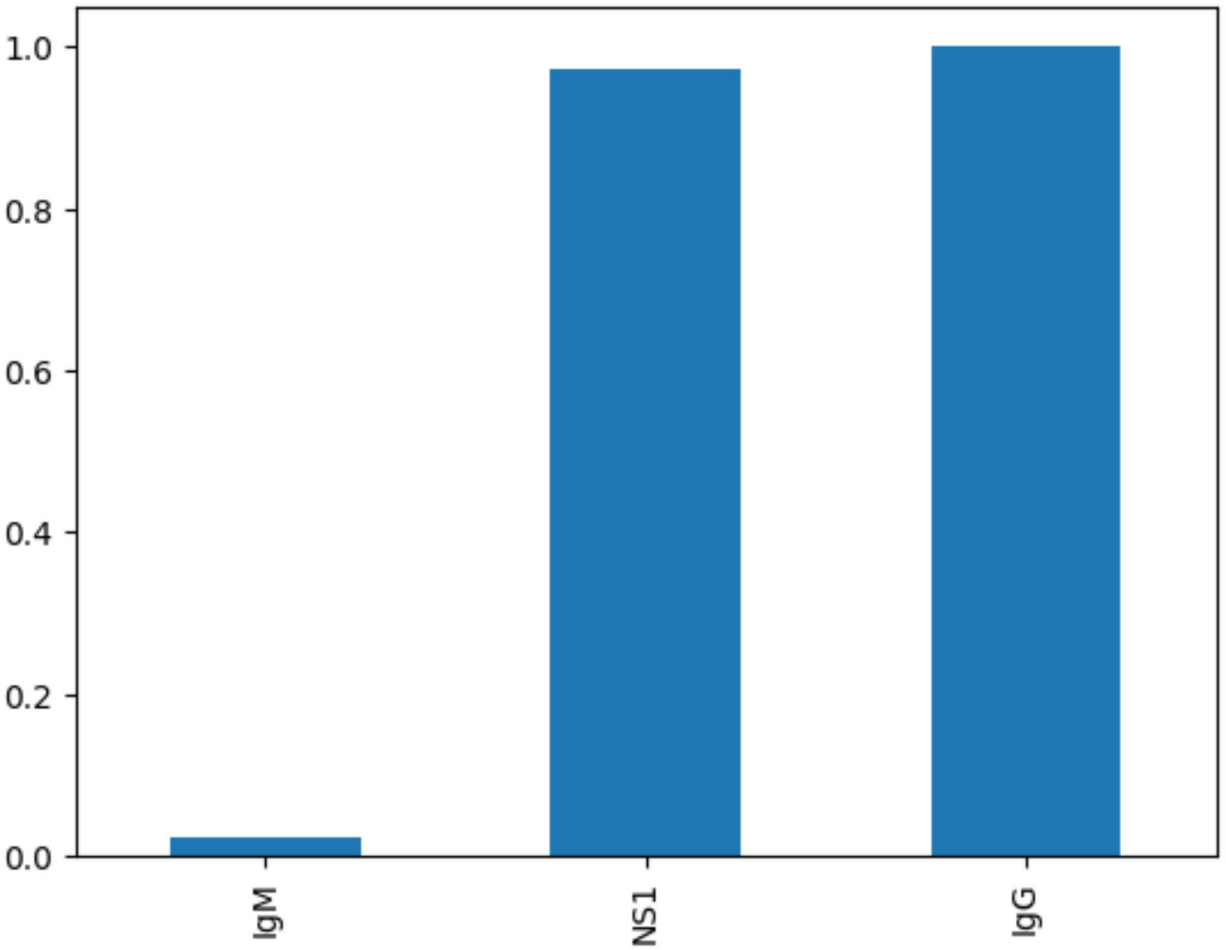
Correlation of all features with the target feature.

The Level-2 diagnosis is mainly based on the serological test results of the probable cases. Level-2 diagnosis is a rule based inference method. Rules has been generated from the unique rows of the dataset, ‘Dengue Dataset of Bangladesh’ from Kaggle^61^. And based on those rules, diagnosis is done in the edge itself and the permanent information is sent to the Cloud. NS1, IgG, IgM are the antigen and antibodies detected in the blood of the person infected with Dengue Virus^62^. This file contains a dengue illness categorical dataset including 1000 responder records with both text and numeric variables. It includes ten columns: Gender, Age, Area, House Type, District, NS1, IgG, IgM, and Outcome. Unwanted features are removed. Only four features which include NS1, IgG, IgM and the outcome has been chosen where the values of all four features were either 0 or 1 (Binary values). The Heatmap for these features has been given below in Fig. 14. The correlation value of each feature with the target feature ‘outcome’ is shown in Fig. 15.

It is observed that there were only 6 unique rows in this dataset. So rules have been formulated using these 6 rows of the dataset to carry out rule-based decision-making. Table 4 given below shows the set of unique rows and below that is the rule generated from the values in the table to get the outcome as 0 (dengue negative) or 1(dengue positive). If the outcome is 1, the dengue is confirmed; otherwise, it is not.

**Table 4:** Unique rows from the dataset with the generated rules.

	NS1	IgG	IgM	Outcome
1	0	0	0	0
2	0	0	1	0
3	1	1	0	1
4	1	1	1	1
5	0	1	0	1
6	0	1	1	1

Generated Rules:

If NS1 = 0 AND IgG = 0 AND IgM in [0, 1], THEN Outcome = 0.

If NS1 in [1, 0] AND IgG = 1 AND IgM in [0, 1], THEN Outcome = 1.

The algorithm used to generate code from the unique values of the table is given below.

**Algorithm 1:**
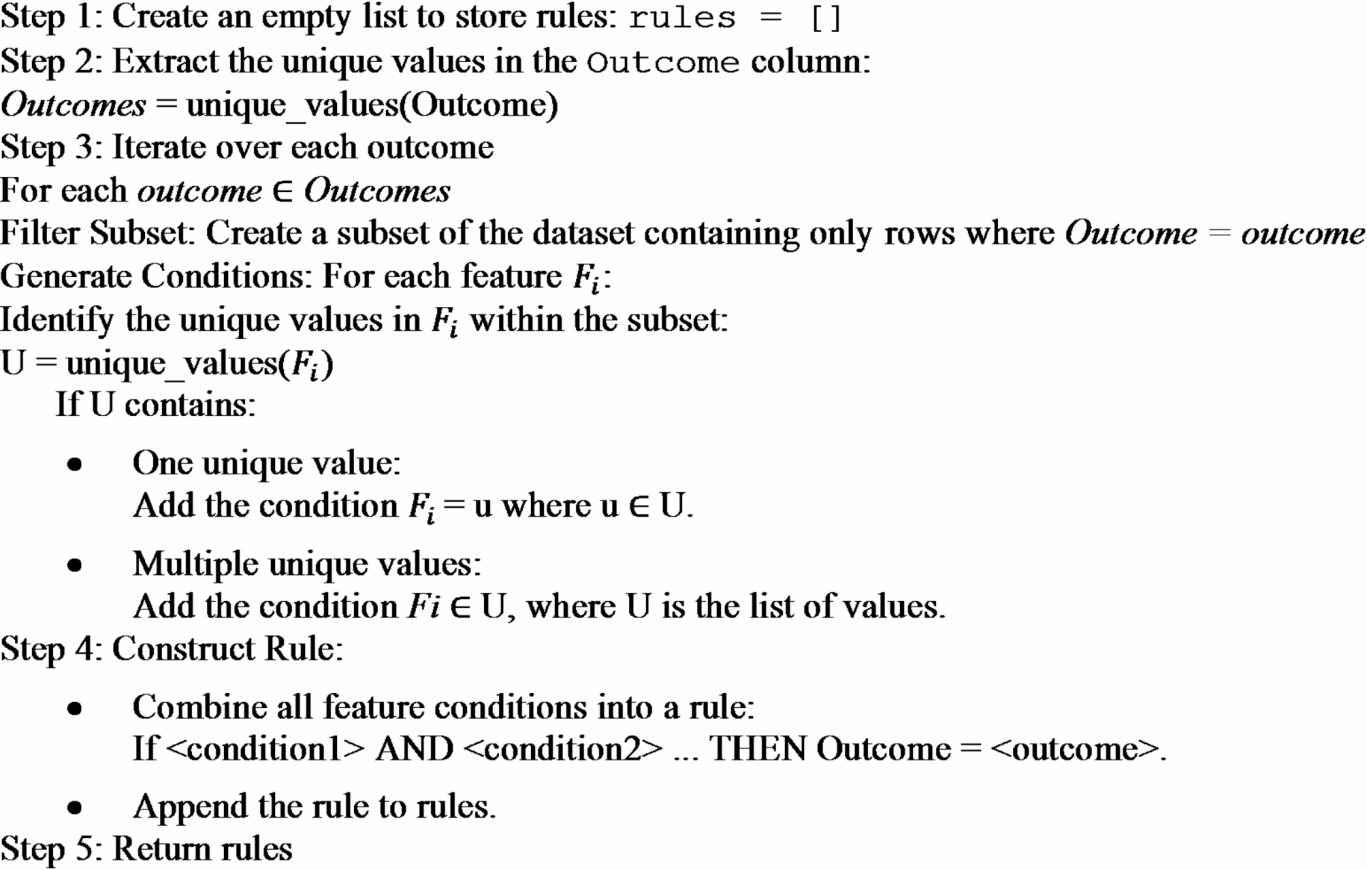
Algorithm used for generating Rules.

Algorithm 1 above generates rules from the 6 unique rows for the rule-based inference method carried out in the edge layer. These rules confirm dengue cases based on the serological test results. The key parameters of this algorithm are:

- Outcome column (Outcome) – The target variable from which unique values (classes) are extracted.
- Features (F = {F₁, F₂, …, F_n_}) – The independent variables used to define conditions in the rules.
- Unique values in the Outcome column (Outcomes) – The distinct classes in the target variable.
- Unique values in each feature (U) – The unique values in each feature within the subset filtered by a specific outcome.
- Generated rules (rules) – A list storing classification rules in the format: IF < conditions > THEN Outcome = < outcome> \text{IF } < \text{conditions}> \text{ THEN Outcome = < outcome>}IF < conditions > THEN Outcome = < outcome>.
- Subset of the dataset – A filtered dataset where Outcome = outcome for each iteration.
- Condition type (Equality or Membership):
  - If a feature has one unique value, the rule uses equality (F_i_ = u).
  - If a feature has multiple unique values, the rule uses set membership (F_i_ ∈ U).

## Discussion

**Fig. 16:**
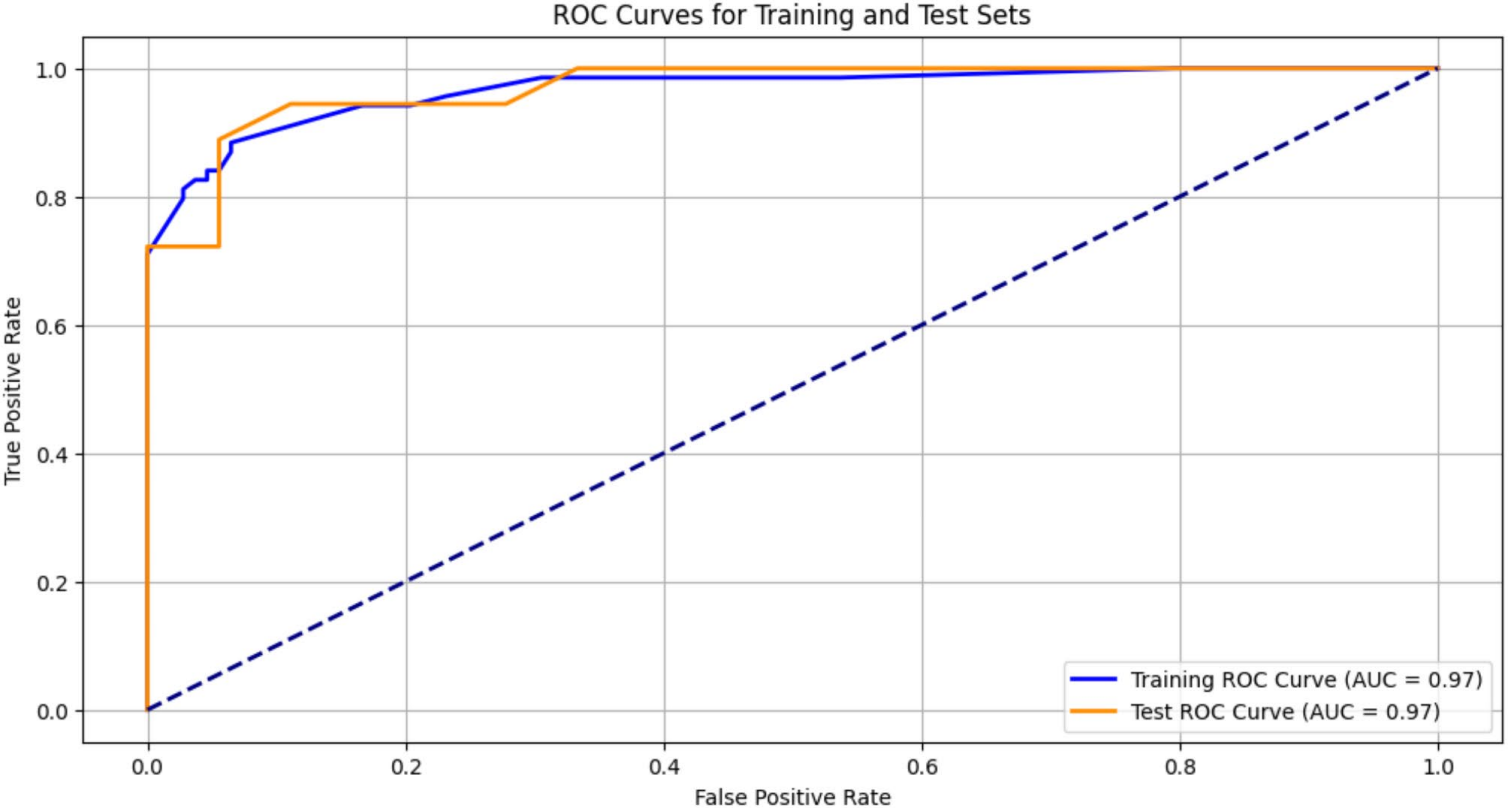
ROC Curve for both training and testing phase of the proposed model.

Various techniques like pre-processing, feature reduction, K-fold cross-validation, hyperparameter tuning, batch normalization and dropout have contributed to the efficiency of the proposed MLP model. The MLP model without using the regularization and dropout techniques produces a F1 score of 97%, a recall of 94%, a specificity of 100%, an accuracy of 97%, etc. The dataset used is a small dataset with 177 rows. These results show evidently that overfitting had occurred. To overcome these problems, techniques like regularization and dropout were used. After which it gave a stabilized result with an accuracy of 92%, F1 score of 90%, recall of 83%, specificity of 100%, and precision of 100%. Further to check whether overfitting has occurred, the ROC curve for both the training phase and the testing phase has been generated. It is shown in Fig. 16. There was no great variation in the AUC (area under curve) value. The model exhibits similar results on both test and train datasets, as evidenced by the AUC of 0.97 for both the training and test sets. Overfitting would be indicated if the AUC scores varied significantly. The ROC curves for the training and test sets have quite similar shapes which is excellent and indicates strong model performance.

From the observation, the accuracy has been improved from 49 to 92% which is evident in Table 3. In most of the previous studies, only level-1 diagnosis is given importance. But it is a fact that only probable cases are found based on level-1 diagnosis and confirmed cases are found after level-2 diagnosis which adds more value to this study. The above ROC curve is an evidence for the balanced functioning of the model. Tsair-Wei_Chein et al.^41^ have used the same dengue dataset in their study. But comparatively our proposed model with the proposed techniques performed well and is found to be stable. The proposed model is expected to perform even better, if the size of the dataset used is improved.

**Table 5:** Comparison between our study and the recent studies.

Paper	Proposed Model /Method	Accuracy	AUC	Fog Framework	XAI Used	Symptom based Diagnosis	Serological Test based Diagnosis
^57^	ANN	86%	NA	No	No	No	No
^10^	Ensemble model	NA	79%	No	No	Yes	No
^12^	Fuzzy Cognitive Map	89.4%	NA	No	No	Yes	Yes
^24^	Tree based classifier	90%	NA	No	No	Yes	No
**Our Study**	**Proposed model**	**92%**	**97%**	**Yes**	**Yes**	**Yes**	**Yes**

Table 5 gives a clear overview of the techniques and methods used in our study and how our study makes the overall dengue diagnosis more efficient. AUC denotes Area Under Curve, XAI denotes Explainable AI, NA denotes Not available in the above table. From Table 5 it is evident that our study not only deals with the diagnosis but also concentrates on the explainability as well as speedy diagnosis by utilizing current technologies like XAI and fog computing. It is very important to know the reason behind an outcome. XAI makes the process easier by explaining every outcome of the model, especially for a black box model like MLP, it adds more value. The professional not only knows the results of the diagnosis but also the reason or cause behind it which is very important for the medical diagnosis. From Table 5 it is also evident that most of the studies have stopped with level-1 diagnosis which is based on symptoms. Our study also emphasizes the level-2 diagnosis only in which the cases are confirmed. Since Level-2 diagnosis deals with fewer features, it employs simplified rule-based inference. The number of people detected during a disease outbreak is enormous. Therefore, controlling it on the edge is challenging. For this reason, we are managing the incoming load with a fog layer. The number of probable cases is comparatively low during the second phase of the diagnosis. So it is carried out in the edge layer. Large volumes of permanent data are moved and stored in the cloud where they may eventually be utilized as diagnostic knowledge.

## Potential challenges in deployment and real-time processing

Compared to cloud servers, fog nodes usually have less processing power (e.g., embedded CPUs, GPUs, or TPUs). To speed up the computation, effective hardware acceleration is needed. Computation time can be decreased by optimizing batch processing and quantizing models. Also data sent to fog nodes could be vulnerable to intrusions. Using differential privacy and encryption strategies can help reduce hazards. In order to deploy our proposed model in a fog computing environment, limitations in memory, processing power, and network latency must be addressed. Real-time processing becomes increasingly viable with the help of performance-enhancing strategies like hardware acceleration, federated learning, and model quantization. High computational cost, energy constraints, network congestion and synchronisation issues are some of the anticipated challenges while working in a fog computing environment. Techniques like model pruning, quantisation, hardware acceleration can be adapted to reduce high computational costs. Edge caching, compression, and batch update techniques can also be employed to overcome network congestion. To overcome the energy constraints problem, lightweight models and adaptive learning rates can be employed. To overcome the synchronisation issues, federated learning and local model update techniques can be employed.

## Limitations of the study

The proposed model has not been tested with diverse datasets. Only one particular dataset has been used to train and test the proposed model. Also there is a need for a large dataset to improve the learning of the model. The dual level disease diagnosis framework should be tested in future in a test bed for fog computing. This would aid in assessing the scalability, effectiveness, and viability of the system in the actual world. Additionally, the dual level diagnosis is not entirely automatic. A health care provider should be on the cutting edge of things. Steps should be developed in the future to make it completely automated. There are a number of restrictions and difficulties when expanding a dual-level diagnosis system to new regions or diseases. Disparities in technology, healthcare infrastructure, and data availability are the primary root causes of these problems. Paper-based records may be used in low-resource situations; hence digitization is necessary before implementation. Strict guidelines on data transmission and storage are enforced by laws like HIPAA (Health Insurance Portability and Accountability Act) (USA) and GDPR (General Data Protection Regulation) (Europe) which is also a limitation. Federated learning approaches can be necessary for cross-border deployment in order to train models without centralizing sensitive data. In developing countries with outdated technology, fog computing environments may not have enough local processing power. Real-time diagnosis can be slowed down by the absence of specialised AI accelerators (such as GPUs and TPUs). Cloud-assisted processing may be impacted by inconsistent or low-bandwidth internet connectivity in rural areas. In areas with inadequate telecommunications infrastructure, network congestion may cause a delay in real-time diagnosis. Different diseases require distinct feature extraction methods, which increases the computing requirements. So, disease-specific model tuning is required. A model trained for a particular disease may not generalize well to diagnosing infectious diseases or neurological disorders. Costs associated with storage, processing, and maintenance rise when scaling to several diseases and geographical areas.

## Conclusion and future work

Various ML and DL techniques have been analysed and evaluated in this study. A three-layered framework comprising of edge, fog and cloud along with the dual-level diagnosis for dengue has been proposed in this study. Some of the contributions of the study are optimised and normalised lightweight MLP model for diagnosing the probable dengue cases in the fog computing environment, the application of explainable AI to enhance the explainability of the proposed MLP model, rule-based inference for the diagnosis of the confirmed cases based on serological test results, and a framework for dual-level diagnosis of dengue that mimics the real-world scenario. By providing real-time location-aware and delay-sensitive services, the integration of fog computing ensures seamless healthcare service delivery from any location at any time. Real-time alerts, efficient use of resources, quick access to medical data, and service quality assurance are just a few advantages of integrating fog computing with healthcare systems. Fog computing is employed as the intermediary layer in the suggested system due of these important advantages. The integrated architectures provide trustworthy patient status reporting, and the application of artificial intelligence techniques can result in accurate disease diagnosis. To improve dependability, we have combined AI with human-in-the-loop verification (i.e., healthcare professional in the edge), as in a few cultures they depend on human beings more than AI. Also, most of the existing works concentrated on first level of the diagnosis which helps in finding only the probable cases but confirmation of the disease is done based on the serological testresults. Also, there is a need for a lightweight model in the fog computing environment. These problems have been addressed in our proposed framework. Level-1 diagnostic is based on patient symptoms that are transferred from the edge layer to the fog. Level-1 diagnostics are performed in the fog to address the storage and computation issues. This study presents an optimized and normalized lightweight MLP (ONL-MLP) along with pre-processing and feature reduction techniques for the Level-1 Diagnosis in the fog computing environment. Compared to conventional MLP models, proposed ONL-MLP uses fewer layers and parameters, which drastically lowers the computational cost. This makes it appropriate for devices with low processing power, including edge nodes in a fog network. To stabilise training, particularly in decentralised networks with varying resources, ONL-MLP incorporates batch normalisation. By normalising input to each layer, this method accelerates convergence and enhances the overall effectiveness of the model under variable conditions. Because data might occasionally be sparse or noisy, ONL-MLP avoids overfitting on small datasets by employing dropout as a regularisation strategy. Hyperparameters like batch size, dropout rates, learning rates, number of layers, and number of neurons in each layer can significantly affect the accuracy and computing efficiency of the ONL-MLP model. Grid Search optimisation has been utilised to get the optimal collection of hyperparameters for the suggested model. It is a methodical search algorithm that examines several possible combinations. Level-2 diagnosis is based on rule-based inference and it is carried out in the edge layer. To support the diagnosis, Explainable AI (XAI) has been used along with the model in Level-1 diagnosis. The proposed model has been compared with other ML/DL models and proved to be more efficient than the other models with an outstanding accuracy of 92% and AUC of 97% for a small dataset. The stability of the model has been proved using the ROC curve for both the testing and training phases. Both the training and test curves show an AUC (Area Under the Curve) of 0.97, which is excellent and indicates strong model performance. The training and test curves closely overlap, suggesting that the model generalizes well and is not overfitting the training data. For the first time, many novel techniques were incorporated in this study. This proposed system analyzes and identifies individuals with dengue by mimicking the actions of a real medical professional. It also satisfies Human-in-the-loops concept, which allows healthcare professionals to validate the results.

In the future, the proposed model should be trained and tested using a large dataset which may increase the efficiency of the model further. Also, in the future, the proposed fog framework should be tested in a real-time scenario. Effective feature engineering techniques can be adapted to increase the performance of the model. We can implement transfer learning from models trained on related infectious diseases to improve performance on dengue. Also, we can apply federated learning, using which multiple hospitals or regions can train the model without sharing sensitive patient data. This approach enhances model robustness while maintaining data security and compliance with privacy regulations (e.g., HIPAA, GDPR). Multimodal data fusion can be employed in order to increase the predictive accuracy. We can include environmental data, image data, and wearable sensor data along with the clinical data. In the future, advanced models like RNN (Recurrent Neural Network) and Transformer models can be used to track the progression of the disease over time. A hybrid deep learning model can be developed by integrating the proposed model with the CNN or any other DL model to perform additional functionalities like analyzing the skin rashes using images. For security reasons, blockchain technology can be integrated. Smart contracts can automate data-sharing agreements between healthcare institutions.

## Data Availability

Two datasets have been used in the current study. The data analysed during the Level-1 phase of diagnosis is from this published article, [https://mhealth.jmir.org/2019/5/e11461/] which is an open access article that permits unrestricted use, distribution, and reproduction of its contents in any medium with citation of the original article along with the copyright information. The datasets used during the Level-2 phase of diagnosis is publicly available dataset from Kaggle, [https://www.kaggle.com/datasets/kawsarahmad/dengue-dataset-bangladesh].
